# Transferrin receptor 1-mediated iron uptake regulates bone mass in mice via osteoclast mitochondria and cytoskeleton

**DOI:** 10.7554/eLife.73539

**Published:** 2022-06-27

**Authors:** Bhaba K Das, Lei Wang, Toshifumi Fujiwara, Jian Zhou, Nukhet Aykin-Burns, Kimberly J Krager, Renny Lan, Samuel G Mackintosh, Ricky Edmondson, Michael L Jennings, Xiaofang Wang, Jian Q Feng, Tomasa Barrientos, Jyoti Gogoi, Aarthi Kannan, Ling Gao, Weirong Xing, Subburaman Mohan, Haibo Zhao

**Affiliations:** 1 Southern California Institute for Research and Education Long Beach United States; 2 Department of Orthopedics, The Third People’s Hospital of Hefei, Third Clinical College, Anhui Medical University Hefei China; 3 Center for Osteoporosis and Metabolic Bone Diseases, Division of Endocrinology, Department of Internal Medicine, University of Arkansas for Medical Sciences Little Rock United States; 4 Department of Orthopedic Surgery, Kyushu University Hospital Fukuoka Japan; 5 Department of Orthopedics, First Affiliated Hospital, Anhui Medical University Hefei China; 6 Division of Radiation Health, Department of Pharmaceutical Sciences, University of Arkansas for Medical Sciences Little Rock United States; 7 Department of Pediatrics, University of Arkansas for Medical Sciences Little Rock United States; 8 Department of Biochemistry and Molecular Biology, University of Arkansas for Medical Sciences Little Rock United States; 9 Department of Physiology and Cell Biology, University of Arkansas for Medical Sciences Little Rock United States; 10 Department of Biomedical Sciences, Texas A&M University Dallas United States; 11 Department of Orthopedics, Duke University Durham United States; 12 Division of Dermatology, Department of medicine, Long Beach VA Healthcare System Long Beach United States; 13 Musculoskeletal Disease Center, VA Loma Linda Healthcare System Loma Linda United States; Icahn School of Medicine at Mount Sinai United States; Icahn School of Medicine at Mount Sinai United States

**Keywords:** transferrin, transferrin receptor 1, osteoclast, bone resorption, bone remodeling, mitochondria, Mouse

## Abstract

Increased intracellular iron spurs mitochondrial biogenesis and respiration to satisfy high-energy demand during osteoclast differentiation and bone-resorbing activities. Transferrin receptor 1 (Tfr1) mediates cellular iron uptake through endocytosis of iron-loaded transferrin, and its expression increases during osteoclast differentiation. Nonetheless, the precise functions of Tfr1 and Tfr1-mediated iron uptake in osteoclast biology and skeletal homeostasis remain incompletely understood. To investigate the role of Tfr1 in osteoclast lineage cells in vivo and in vitro, we crossed *Tfrc* (encoding Tfr1)-floxed mice with *Lyz2 (LysM)-*Cre and *Cathepsin K* (*Ctsk*)-Cre mice to generate *Tfrc* conditional knockout mice in myeloid osteoclast precursors (Tfr1^ΔLysM^) or differentiated osteoclasts (Tfr1^ΔCtsk^), respectively. Skeletal phenotyping by µCT and histology unveiled a significant increase in trabecular bone mass with normal osteoclast number in long bones of 10-week-old young and 6-month-old adult female but not male Tfr1^ΔLysM^ mice. Although high trabecular bone volume in long bones was observed in both male and female Tfr1^ΔCtsk^ mice, this phenotype was more pronounced in female knockout mice. Consistent with this gender-dependent phenomena, estrogen deficiency induced by ovariectomy decreased trabecular bone mass in Tfr1^ΔLysM^ mice. Mechanistically, disruption of Tfr1 expression attenuated mitochondrial metabolism and cytoskeletal organization in mature osteoclasts in vitro by attenuating mitochondrial respiration and activation of the Src-Rac1-WAVE regulatory complex axis, respectively, leading to decreased bone resorption with little impact on osteoclast differentiation. These results indicate that Tfr1-mediated iron uptake is specifically required for osteoclast function and is indispensable for bone remodeling in a gender-dependent manner.

## Introduction

Bone mass and structure in adults are maintained by constant bone remodeling with balanced bone formation by osteoblasts and bone resorption by osteoclasts (Zaidi, 2007; Siddiqui and Partridge, 2016). Under pathological conditions, however, excessive bone resorption caused by either increased number or exaggerated activities of osteoclasts leads to bone loss which is a hallmark of many metabolic bone diseases such as osteoporosis, rheumatoid arthritis, Paget’s disease of bone, periodontal disease, and tumor bone metastasis (Mbalaviele et al., 2017; Novack and Teitelbaum, 2008). Osteoclasts are multinucleated cells formed by fusion of mononuclear precursors that are differentiated from the monocyte/macrophage lineage of hematopoietic cells. This process is triggered by two indispensable cytokines M-CSF (macrophage colony-stimulating factor) and RANKL (receptor activator of NF-κB ligand) (Walker, 1975; Xiao et al., 2017; Boyle et al., 2003; Nakashima et al., 2012). Upon attachment to bone, osteoclasts organize their actin cytoskeleton to form actin-rings that seal the resorptive microenvironment (Väänänen et al., 2000; Teitelbaum, 2011). The dissolution of bone minerals and digestion of organic bone matrix mainly type I collagen are executed by hydrochloric acid and acidic hydrolase cathepsin K, respectively (Zhao, 2012; Fujiwara et al., 2016). Both osteoclast formation and bone-resorbing activity of mature osteoclasts demand high energy. However, the pathways and molecular mechanisms regulating osteoclast energy metabolism in osteoclasts remain largely unknown.

Both glycolysis and mitochondrial oxidative phosphorylation (OXPHOS) increase during osteoclast differentiation (Kubatzky et al., 2018; Arnett and Orriss, 2018; Kim et al., 2007; Indo et al., 2013; Li et al., 2020a). Osteoclasts contain numerous mitochondria (Ch’uan, 1931; Holtrop and King, 1977). The mitochondrial respiratory complex I and the key mitochondria transcriptional regulator PGC-1β (peroxisome proliferator-activated receptor γ coactivator 1β) are crucial for osteoclast differentiation (Jin et al., 2014; Ishii et al., 2009). Mitochondrial ROS (reactive oxygen species) stimulates osteoclast differentiation (Srinivasan et al., 2010; Kim et al., 2013; Kim et al., 2017). On the contrary, it has been reported that decreased mitochondrial biogenesis and activity by PGC-1β deletion in osteoclast lineage cells disrupt osteoclast cytoskeletal organization and function but not osteoclastogenesis (Zhang et al., 2018). Loss of G-protein Gα13 in osteoclast progenitors promotes mitochondrial respiration and cytoskeleton organization but has little effects on osteoclastogenesis in cultures and in mice (Nakano et al., 2019). Heretofore, the mechanisms by which energy metabolism regulates osteoclast differentiation and function remain unclear.

Iron is a nutritional element that plays a fundamental role in mitochondrial metabolism and the biosynthesis of heme and Fe-S clusters which are critical components of the mitochondrial respiratory complexes (Figure 1—figure supplement 1A and Xu et al., 2013). Both systemic and cellular iron homeostasis play a pivotal role in bone remodeling (Balogh et al., 2018). Osteoporosis and pathological bone fractures are commonly associated with the hereditary iron-overload disease hemochromatosis and the acquired iron-overload conditions in treatment of thalassemia and sickle cell disease (Almeida and Roberts, 2005; Fung et al., 2008; Dede et al., 2016; Jandl et al., 2020). Moreover, increased bone resorption and/or decreased bone formation have been observed in genetic mouse models of iron overload diseases and in mice fed with excessive iron (Tsay et al., 2010; Guggenbuhl et al., 2011; Xiao et al., 2015). In contrast to iron overload, much less is known about the influence of iron deficiency on bone cells and bone homeostasis. Severe iron deficiency impairs both bone resorption and bone formation and causes osteopenia in rats (Katsumata et al., 2009; Diaz-Castro et al., 2012), whereas iron chelation inhibits osteoclastogenesis and bone resorption in vitro and in vivo and attenuates estrogen deficiency induced bone loss in mice (Ishii et al., 2009; Guo et al., 2015).

There are two transferrin (Tf) receptors (Tfrs) in mammalian cells (Kawabata, 2019). Transferrin receptor 1 (Tfr1), encoded by *Tfrc* gene, is ubiquitously expressed with high affinity to Fe^3+^-loaded holo-Tf. Germline deletion of *Tfrc* in mice leads to early embryonic lethality due to severe defects in erythroid and neuronal development (Levy et al., 1999). A missense mutation in *TFRC* causes combined immunodeficiency in human (Jabara et al., 2016). The tissue-specific deletion of *Tfrc* gene in neurons, skeletal muscles, cardiomyocytes, hematopoietic stem cells, and adipocytes in mice disrupts cellular iron homeostasis and causes severe metabolic defects in these tissues (Matak et al., 2016; Barrientos et al., 2015; Xu et al., 2015; Wang et al., 2020; Li et al., 2020b). In contrast to Tfr1, Tfr2 has lower affinity to holo-Tf and is predominantly expressed in liver and erythroid precursor cells (Kawabata, 2019). Mutations of *TFR2* in human and germline or hepatocyte-specific deletion of *Tfr2* in mice cause type 3 hemochromatosis (Camaschella et al., 2000; Fleming et al., 2002). More recently, Tfr2 has been reported to regulate osteoblastic bone formation and bone mass in mice, independent of its function in iron homeostasis (Rauner et al., 2019). Nevertheless, the cell autonomous functions of Tfr1 and Tfr2 in osteoclast lineage cells remain unknown.

To elucidate the role of Tfr1 and Tfr1-mediated iron uptake in osteoclast lineage cells, we generated *Tfrc* conditional knockout mice in myeloid osteoclast precursors and differentiated osteoclasts by crossing *Tfrc*-flox mice with *Lyz2*-Cre and *Ctsk*-Cre mice, respectively, and used them for comprehensive skeletal phenotyping in vivo and mechanistic studies in vitro.

## Results

### Tfr1-mediated iron uptake is the major route for iron acquiring in murine osteoclasts

**Figure 1.**
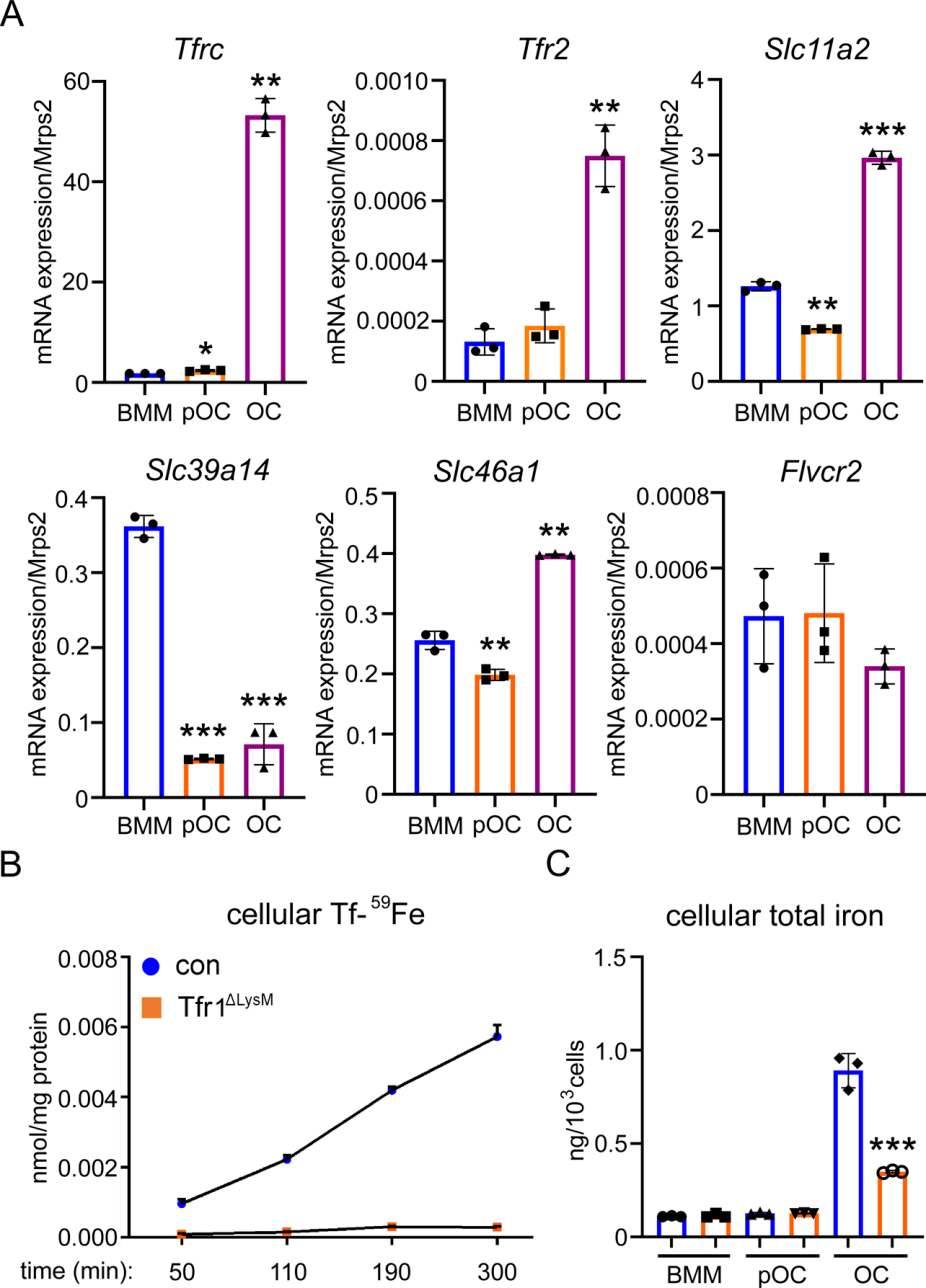
Tfr1 is a major transferrin transporter and regulates cellular iron homeostasis in osteoclasts. (**A**) Detection of mRNA expression of genes involved in iron uptake and export pathways in bone marrow monocytes (BMM), mononuclear pre-osteoclasts (pOC), and mature osteoclasts (OC) by real-time quantitative PCR. *Tfrc* encodes Tfr1; *Slc11a1* encodes Dmt1 (divalent metal ion transporter 1); *Slc39a14* encodes Zip14; *Slc46a1* encodes heme transporter Hcp1; *Flvcr2* encodes Flvcr2 heme transporter 2. (**B**) Measurement of ^59^Fe-labeled transferrin (Tf-^59^Fe) uptake in control (con) and Tfr1 myeloid conditional knockout (Tfr1^ΔLysM^) osteoclasts by a gamma counter. (**C**) Measurement of intracellular total iron by a colorimetric iron assay kit in control and Tfr1-deficient osteoclast lineage cells. The data are presented as mean ± SD. n=3. * p<0.05, ** p<0.01, and *** p<0.001 vs BMM (**A**) and vs control OC (**C**) by one-way ANOVA and Student’s t-test. Figure 1—source data 1.Tfr1 is a major transferrin transporter and regulates cellular iron homeostasis in osteoclasts.

Mammalian cells acquire iron through the uptake of Tf, heme, ferritins, and by non-transferrin bound iron (NTBI) (Figure 1—figure supplement 1B; Pantopoulos et al., 2012). In Tf-dependent pathway, holo-Tf binds to Tfrs on cell surface, and the complex is then internalized via endocytosis. Fe^3+^ is released from Tf in endosomes and is reduced to ferrous iron (Fe^2+^) by the STEAP (six-transmembrane epithelial antigen of the prostate) family of metaloreductases before being transported to the cytoplasm via DMT1 (divalent metal transporter 1) (Gammella et al., 2017). Cellular iron that is not utilized is either stored in ferritins or is exported via ferroportin (FPN) (Donovan et al., 2006; Hentze et al., 2010). In this study, we first set out to determine which of the four cellular iron uptake pathways operate in osteoclasts. For this purpose, we quantified the mRNA level of genes encoding the major transporters along each iron acquiring pathway (Figure 1—figure supplement 1B) by real-time PCR in three different stages of murine osteoclast lineage cells: bone marrow monocytes (BMM), mononuclear osteoclast precursors, and multinucleated mature osteoclasts. By this assay, we observed that the expression of genes participated in Tf/Tfr-mediated iron uptake: *Tfrc*, *Tfr2*, and *Slc11a2* (encoding Dmt1) were upregulated in mature osteoclasts (Figure 1A). We have previously reported that Steap4 is highly expressed in mature osteoclasts and plays an important role in cellular iron homeostasis in osteoclasts in vitro (Zhou et al., 2013). Together, all key mediators of Tf-dependent iron uptake pathway are upregulated during osteoclast differentiation. In contrast, the mRNA expression of *Slc39a14* (encoding Zip14, a transporter mediating cellular iron entrance via NTBI) was decreased during osteoclast differentiation. The expression of *Slc46a1*, which encodes the heme transporter Hcp1, was slightly increased in mature osteoclasts. The mRNA level of *Flvcr2*, which encodes another heme transporter, was low and did not change during osteoclast differentiation (Figure 1A). The mRNAs of *Timd2* and *Scara5*, which encode two plasma membrane transporters of ferritin, were undetectable in osteoclast lineage cells (data not shown).

**Figure 1—figure supplement 1.**
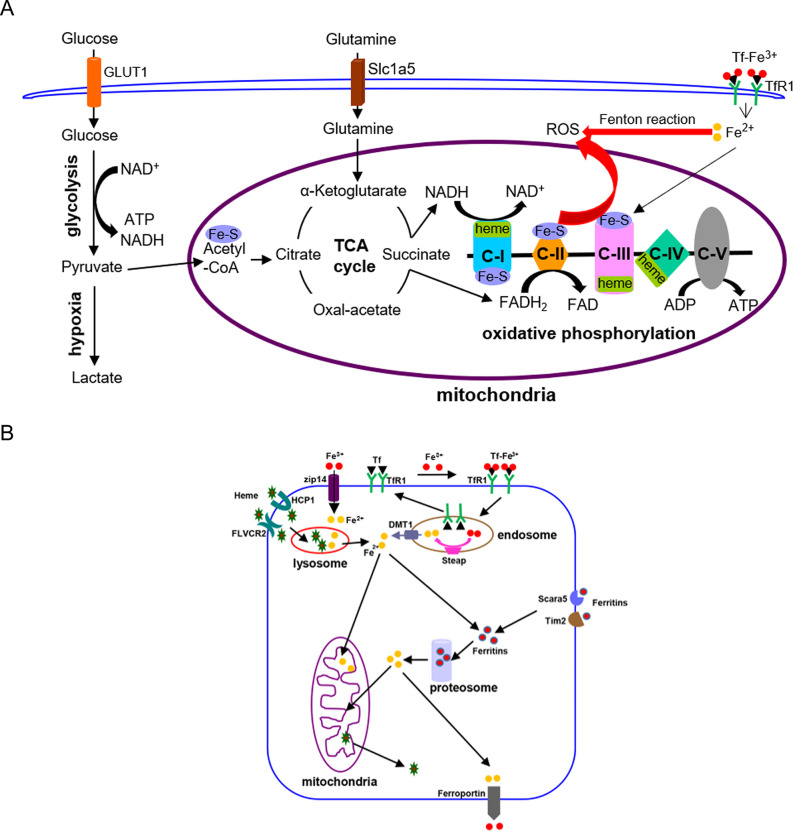
Graphic diagrams of iron in energy metabolism in mammalian cells (**A**) and cellular iron-uptake pathways (**B**). C-I to C-V: mitochondrial respiration chain complex-I to complex-V; DMT1: divalent metal ion transporter 1; GLUT1: glucose transporter 1; ROS: reactive oxygen species; Slc1a5: glutamine transporter; Steap: metalloreductase of the six transmembrane epithelial antigen of the prostate family proteins; Tf: transferrin; TfR1: transferrin receptor 1.

Although both *Tfrc* and *Tfr2* mRNA expression increase during osteoclastogenesis, the level of *Tfr2* mRNA was much lower than that of *Tfrc* (Figure 1A). Moreover, deletion of Tfr1 completely eliminated the uptake of Tf-^59^Fe in mature osteoclasts, indicating that Tfr1 is the predominant receptor for Tf-uptake in osteoclasts (Figure 1B). Despite the previous finding that Tfr2-deletion in monocytes has no effects on iron homeostasis (Rishi et al., 2016), the role of Tfr2 in osteoclast iron uptake and function in vitro and in vivo needs to be further investigated in future. There was a dramatic increase in cellular iron concentration in mature osteoclasts compared to monocytes and pre-osteoclasts. Loss of Tfr1 in osteoclasts led to a >50% decrease in total intracellular iron content (Figure 1C), suggesting that Tf-dependent iron uptake contributes remarkably to iron acquirement in mature osteoclasts. We and others have previously found that the mRNA expression of *Slc40a1* (encoding Fpn, the only iron exporter identified so far in mammalian cells) decreases during osteoclast differentiation (Gu et al., 2015; Wang et al., 2018) and loss of Fpn in myeloid osteoclast precursors increases cellular iron and stimulates osteoclastogenesis in vitro and in vivo (Wang et al., 2018). Therefore, by transcriptional upregulation of Tf-dependent iron uptake pathway and concomitant downregulation of iron exporter Fpn, osteoclasts augment their cellular iron level to meet high energy demand during osteoclast differentiation and bone resorption. Nevertheless, the role of Tfr1 and Tf-dependent iron uptake in osteoclast biology and bone remodeling has not been elucidated using genetically modified mouse models.

### Loss of Tfr1 in myeloid osteoclast precursors increases trabecular bone mass of long bones prominently in adult female mice

The *Tfrc* germline deletion mice are embryonically lethal (Levy et al., 1999). To elucidate the role of Tfr1 in osteoclast differentiation and bone resorption during post-natal bone modeling and remodeling, we generated *Tfrc* myeloid conditional knockout mice on C57BL6/J background by crossing *Tfrc*-flox mice, in which the exons 3–6 of murine *Tfrc* gene were flanked by two loxP sites (Chen et al., 2015), with *Lyz2 (LysM*)-Cre mice. Our pilot study demonstrated that the skeletal phenotypes of three lines of control mice: *Tfrc*^flox/flox^;*Lyz2*^+/+^, Tfrc^+/+^;*Lyz2*^Cre/Cre^, and *Tfrc*^+/+^;*Lyz2*^+/+^ male and female mice were indistinguishable (Figure 2—figure supplement 1). We also noticed that single allele of *Lyz2-Cre* (*Tfrc*^flox/flox^;*Lyz2*^Cre/+^) only led to partial deletion of Tfr1 in osteoclast lineage cells, whereas two copies of *Lyz2-Cre* (*Tfrc*^flox/flox^;*Lyz2*^Cre/Cre^) completely eliminated Tfr1 in osteoclasts detected by western blotting (see Figure 4C). Moreover, trabecular bone mass and structure in *Tfrc*^flox/flox^;*Lyz2*^Cre/+^ mice evaluated by µCT were similar to their gender-matched littermate control mice (Figure 2—figure supplement 2). Therefore, we used *Tfrc*^flox/flox^;+/+ and *Tfrc*^flox/flox^;*Lyz2*^Cre/Cre^ mice as control and Tfr1 myeloid conditional knockout (Tfr1^ΔlysM^) mice, respectively, for further in vivo and in vitro studies.

Both male and female Tfr1^ΔlysM^ mice were born at expected Mendelian ratio and developed normally with similar body size and weight compared to their littermate controls at 3 weeks, 10 weeks, and 6 months ages (data not shown). The serum levels of total iron and iron-regulating hormone hepcidin as well as the number of red blood cells in circulation of male and female Tfr1^ΔlysM^ mice were similar compared to their respective controls (Figure 2—figure supplement 3), indicating that loss of Tfr1 in myeloid lineage cells has no effects on systemic iron homeostasis and erythropoiesis.

**Figure 2.**
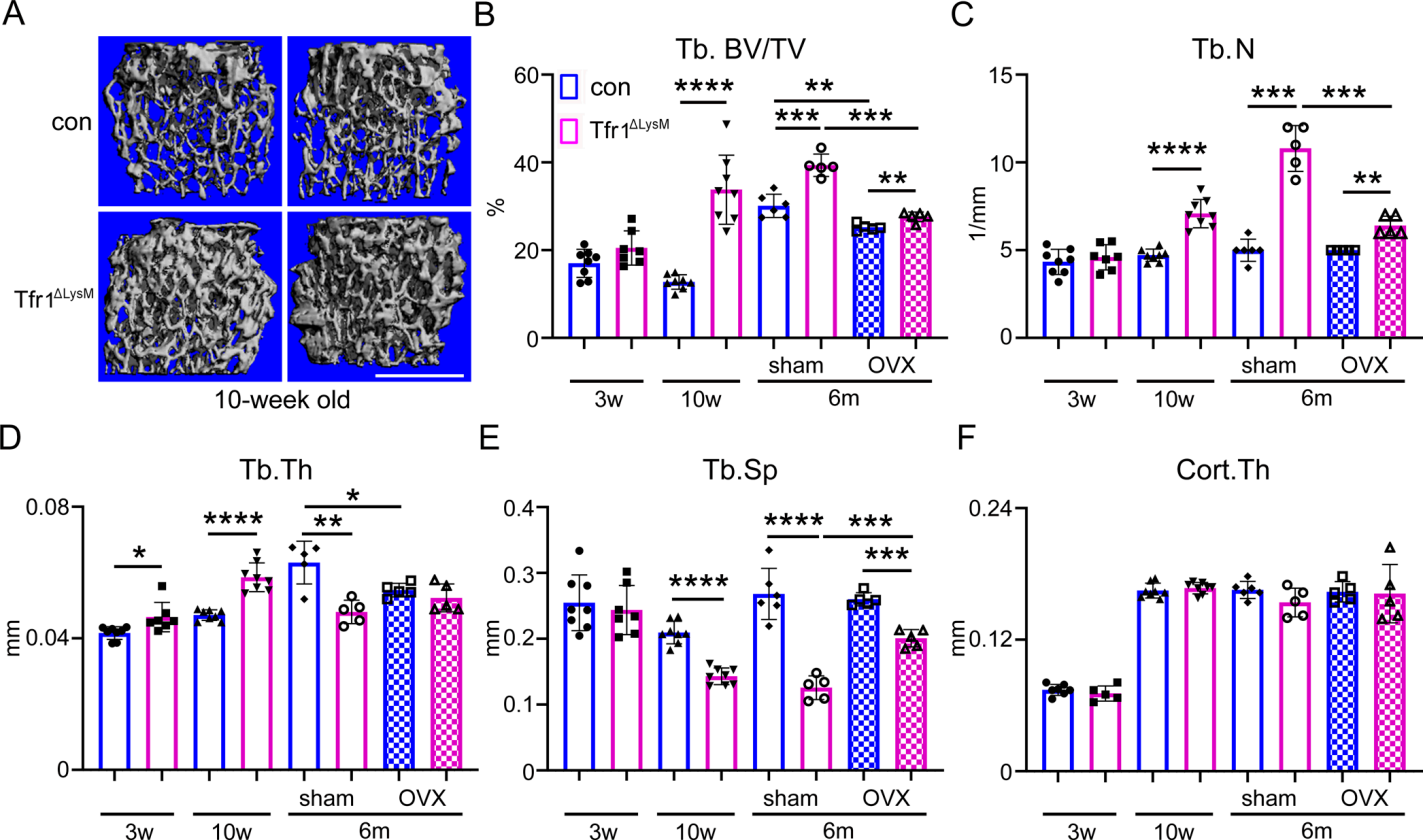
*Tfr1* myeloid conditional knockout female mice develop normally and display increased trabecular bone mass in femurs at 10-week-old and 6-month-old ages. (**A**) Representative µCT images of distal femurs of 10-week-old female control (con) and Tfr1^ΔLysM^ mice. scale bar = 1 mm. (**B– F**) µCT analysis of trabecular and cortical bone mass and structure of distal femurs isolated from three different ages of female con and Tfr1^ΔLysM^ mice. Tb: trabecular bone; BV/TV: bone volume/tissue volume; Tb.N: trabecular number; Tb.Th: trabecular thickness; Tb.Sp: trabecular spacing; Cort.Th: cortical bone thickness. The data are presented as mean ± SD. n=5–13. * p<0.05; ** p<0.01; *** p<0.001; **** <0.0001 by one-way ANOVA. Figure 2—source data 1.*Tfr1* myeloid conditional knockout female mice develop normally and display increased trabecular bone mass in femurs at 10-week-old and 6-month-old ages.

The µCT analysis of distal femurs isolated from 3-week-old control, and Tfr1^ΔlysM^ mice revealed that loss of Tfr1 in osteoclast myeloid precursor cells at pre-pubertal age led to a mild increase in trabecular bone mass (bone volume/tissue volume [BV/TV]), trabecular number (Tb.N), and a decrease in trabecular spacing (Tb.Sp) in male Tfr1^ΔlysM^ mice compared to gender matched control mice. Only a slight increase in trabecular thickness (Tb.Th) was observed in pre-pubertal female Tfr1^ΔlysM^ mice compared to control mice (Figure 2 and Figure 2—figure supplement 4). At 10-week post-pubertal age, however, trabecular BV/TV (>twofold), Tb.N, and Tb.Th were increased while Tb.Sp was deceased in the distal femurs of female Tfr1^ΔlysM^ mice compared to controls. The increased trabecular bone mass phenotype in female Tfr1^ΔlysM^ mice was retained at 6 months of age (Figure 2A-E). There was no difference in any of the above trabecular bone parameters between control and Tfr1^ΔlysM^ male mice at either 10 weeks or 6 months age (Figure 2—figure supplement 4A–4E). The trabecular bone mass was significantly higher in male control mice after puberty. Since increased trabecular bone volume only occurred in Tfr1-deficient female mice, the trabecular bone mass of Tfr1^ΔlysM^ female mice at 10 week and 6 month of ages was similar to age-matched control as well as Tfr1^ΔlysM^ male mice. No changes in the cortical bone thickness (Cort.Th) were detected by µCT in the mid-shaft of femurs of both male and female Tfr1^ΔlysM^ mice at all three ages compared to their respective controls (Figure 2F and Figure 2—figure supplement 4F). The µCT analysis of tibias showed similar trabecular bone mass increase in female but not male Tfr1^ΔlysM^ mice (data not shown). By contrast, a moderate increase in trabecular bone mass of vertebral bones was observed in 6-month-old, but not 10-week-old, Tfr1^ΔlysM^ female mice (Figure 2—figure supplement 5). Again, the vertebral bone mass of 6-month-old control and Tfr1^ΔlysM^ male mice was indistinguishable (data not shown). Taken together, these results indicate that the effects of Tfr1-deletion in osteoclast precursor cells on trabecular bone mass in post-pubertal and adult mice are gender- and site-dependent. To determine if loss of Tfr1 in osteoclasts affects ovariectomy (OVX)-induced bone loss, control and Tfr1^ΔLysM^ female mice at 4.5 months of age were ovariectomized to mimic human post-menopausal osteoporosis and skeletal phenotype was determined at 6 months of age. While OVX induced bone loss in Tfr1^ΔLysM^ mice, the trabecular bone mass of Tfr1^ΔLysM^ mice was significantly higher than control mice after OVX.

**Figure 2—figure supplement 1.**
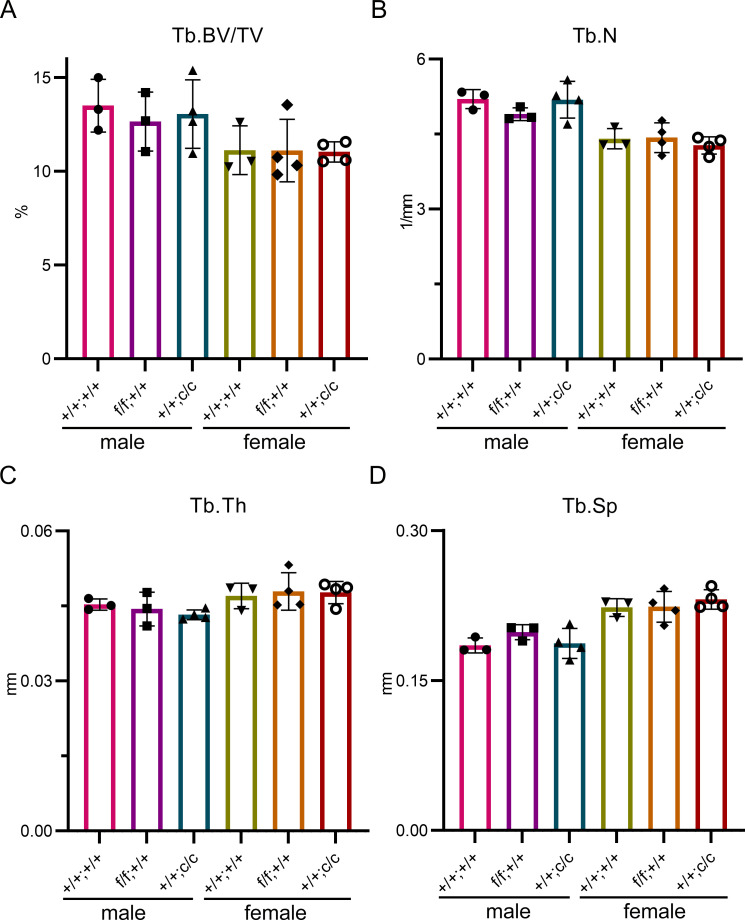
The trabecular bone mass and structure among three different genotypes of control mice are similar. μCT analysis of distal femurs of three different genotypes of 10-week-old male and female control mice. Tb: trabecular bone; BV/TV: bone volume/tissue volume; Tb.N: trabecular number; Tb.Th: trabecular thickness; Tb.Sp: trabecular spacing. The data are presented as mean ± SD and analyzed by one-way ANOVA. n=3–4. Figure 2—figure supplement 1—source data 1.The trabecular bone mass and structure among three different genotypes of control mice are similar.

**Figure 2—figure supplement 2.**
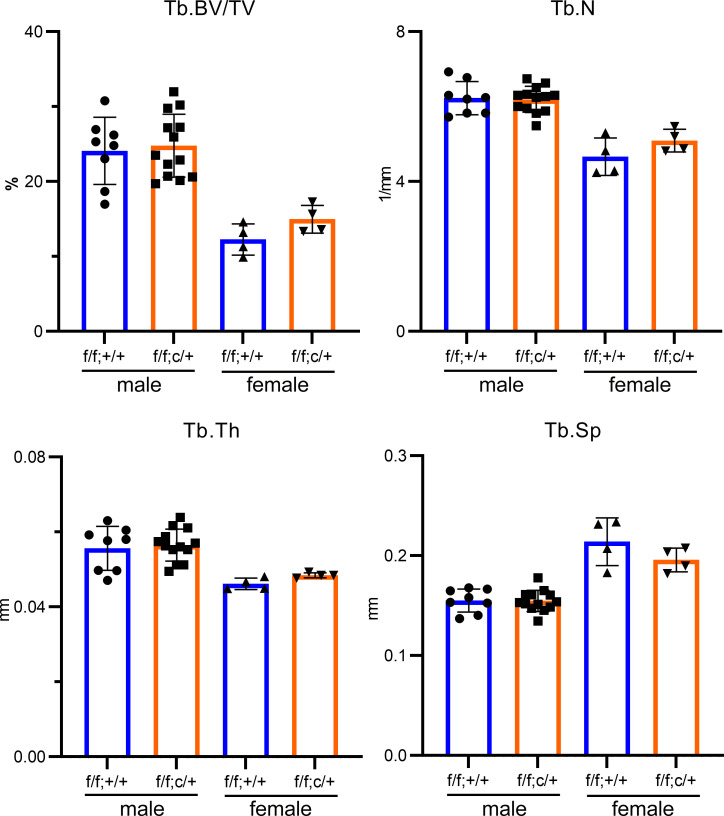
Partial deletion of Tfr1 by one-allele of LysM-Cre has no effects on bone mass and structure in male and female mice on C57BL6/J background. µCT analysis of trabecular bone mass and structure of distal femurs from 10-week-old C57BL6/J background mice. Tb: trabecular bone; BV/TV: bone volume/tissue volume; Tb.N: trabecular number; Tb.Th: trabecular thickness; Tb.Sp: trabecular spacing. The data are presented as mean ± SD and analyzed by Student’s t-test. n=4–13. Figure 2—figure supplement 2—source data 1.Partial deletion of Tfr1 by one-allele of LysM-Cre has no effects on bone mass and structure in male and female mice on C57BL6/J background.

**Figure 2—figure supplement 3.**
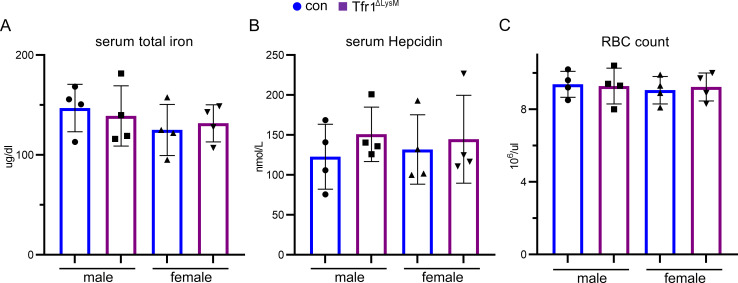
Loss of Tfr1 in myeloid osteoclast precursor cells did not alter the serum iron and hepcidin levels and had no effects on erythropoiesis in 10-week-old male and female mice. The serum total iron concentration of 10-week-old control (con) and Tfr1^ΔLysM^ mice was measured by a colorimetric based iron detection kit from Fisherscientic Inc. The serum hepcidin level was measured by a Hepcidin Murine-Complete ELISA kit from Intrinsic Lifesciences. The red blood cell number of peripheral bloods was counted using hemacytometer. The data are presented as mean ± SD and analyzed by one-way ANOVA. n=4. Figure 2—figure supplement 3—source data 1.Loss of Tfr1 in myeloid osteoclast precursor cells did not alter the serum iron and hepcidin levels and had no effects on erythropoiesis in 10-week-old male and female mice.

**Figure 2—figure supplement 4.**
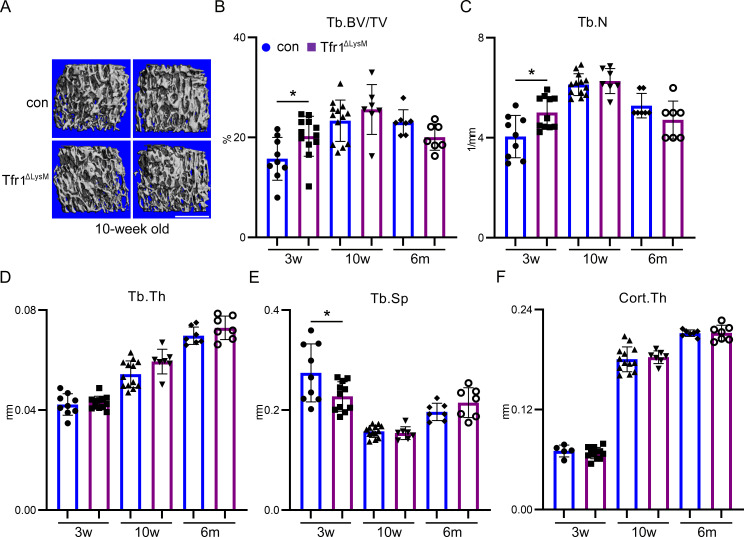
*Tfr1* myeloid conditional knockout male mice develop normally and exhibit no significant changes in trabecular and cortical bone. (**A**) Representative µCT images of distal femurs of 10-week-old male control (con) and Tfr1^ΔLysM^ mice. scale bar = 1 mm. (**B–F**) µCT analysis of trabecular and cortical bone mass and structure of distal femurs from 3-week-old, 10-week-old, and 6-month-old of male con and Tfr1^ΔLysM^ mice. Tb: trabecular bone; BV/TV: bone volume/tissue volume; Tb.N: trabecular number; Tb.Th: trabecular thickness; Tb.Sp: trabecular spacing; Cort.Th: cortical bone thickness. The data are presented as mean ± SD. n=5–13. * p<0.05 vs con by Student’s t-test. Figure 2—figure supplement 4—source data 1.*Tfr1* myeloid conditional knockout male mice develop normally and exhibit no significant changes in trabecular and cortical bone.

**Figure 2—figure supplement 5.**
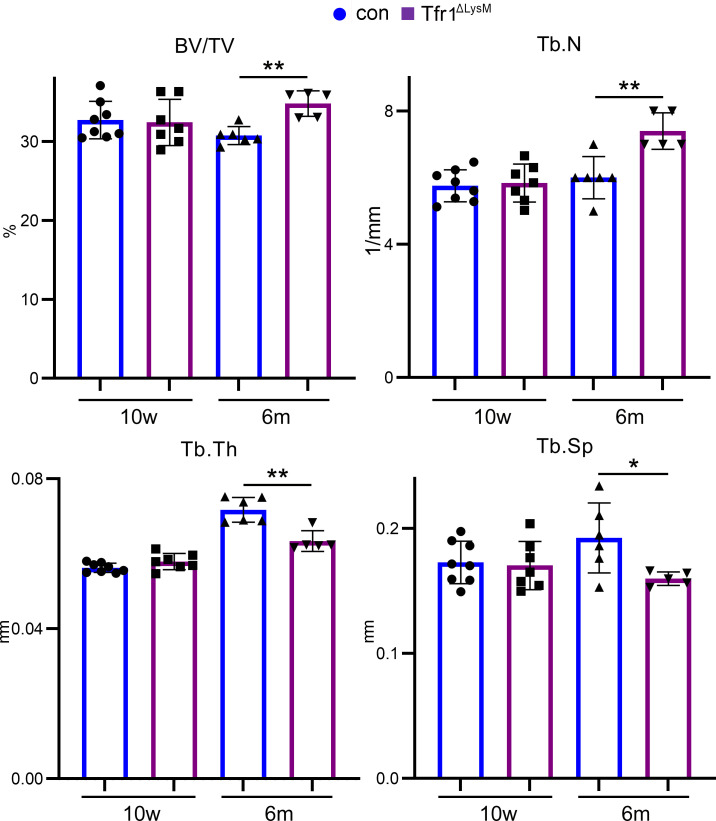
Loss of Tfr1 in myeloid osteoclast precursor cells had less effects on trabecular bone mass in lumber vertebrae than femurs. µCT analysis of lumber vertebrae of 10-week-old and 6-month-old control (con) and Tfr1^ΔLysM^ mice. Tb: trabecular bone; BV/TV: bone volume/tissue volume; Tb.N: trabecular number; Tb.Th: trabecular thickness; Tb.Sp: trabecular spacing; BMD: bone mineral density; Cort: cortical bone. The data are presented as mean ± SD. n=5–8. * p<0.05; ** *P*<0.01 vs con by one-way ANOVA. Figure 2—figure supplement 5—source data 1.Loss of Tfr1 in myeloid osteoclast precursor cells had less effects on trabecular bone mass in lumber vertebrae than femurs.

**Figure 2—figure supplement 6.**
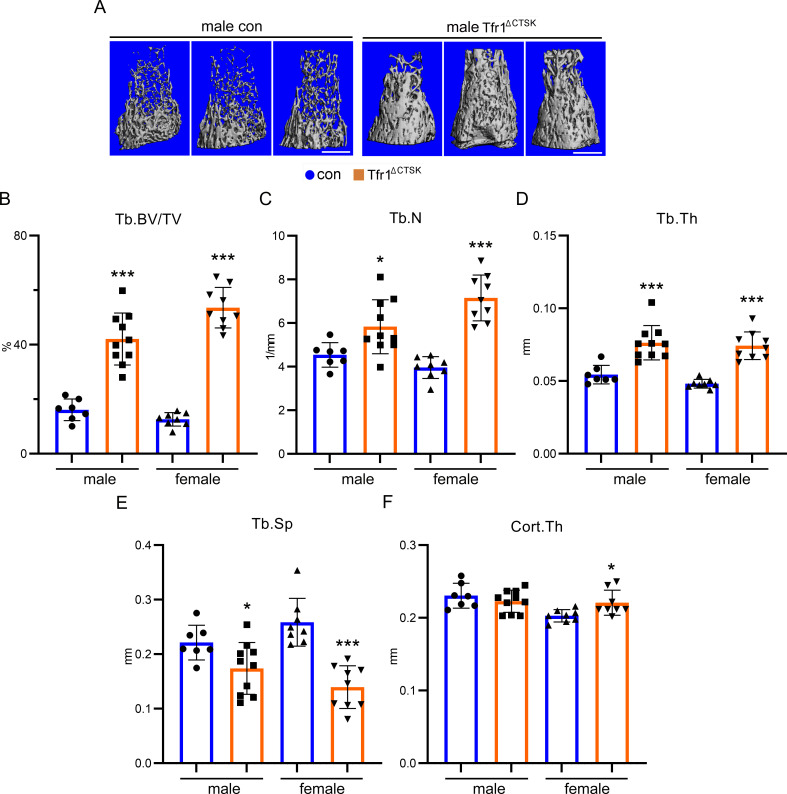
Deletion of *Tfr1* in cathepsin K-Cre expressing osteoclasts results in increased trabecular bone mass in distal femurs of 10-week-old male and female mice. (**A**) The representative µCT images of distal femurs of male and female control (con) and Tfr1^ΔCTSK^ mice on the 129×C57BL6J mixed background. scale bar = 1 mm. (**B–F**) µCT analysis of trabecular and cortical bone mass and structure of distal femurs. Tb: trabecular bone; BV/TV: bone volume/tissue volume; Tb.N: trabecular number; Tb.Th: trabecular thickness; Tb.Sp: trabecular spacing; Cort: cortical bone. The data are presented as mean ± SD. n=7–10. * p<0.05, *** p<0.001 vs con by one-way ANOVA. Figure 2—figure supplement 6—source data 1.Deletion of *Tfr1* in cathepsin K-Cre expressing osteoclasts results in increased trabecular bone mass in distal femurs of 10-week-old male and female mice.

**Figure 2—figure supplement 7.**
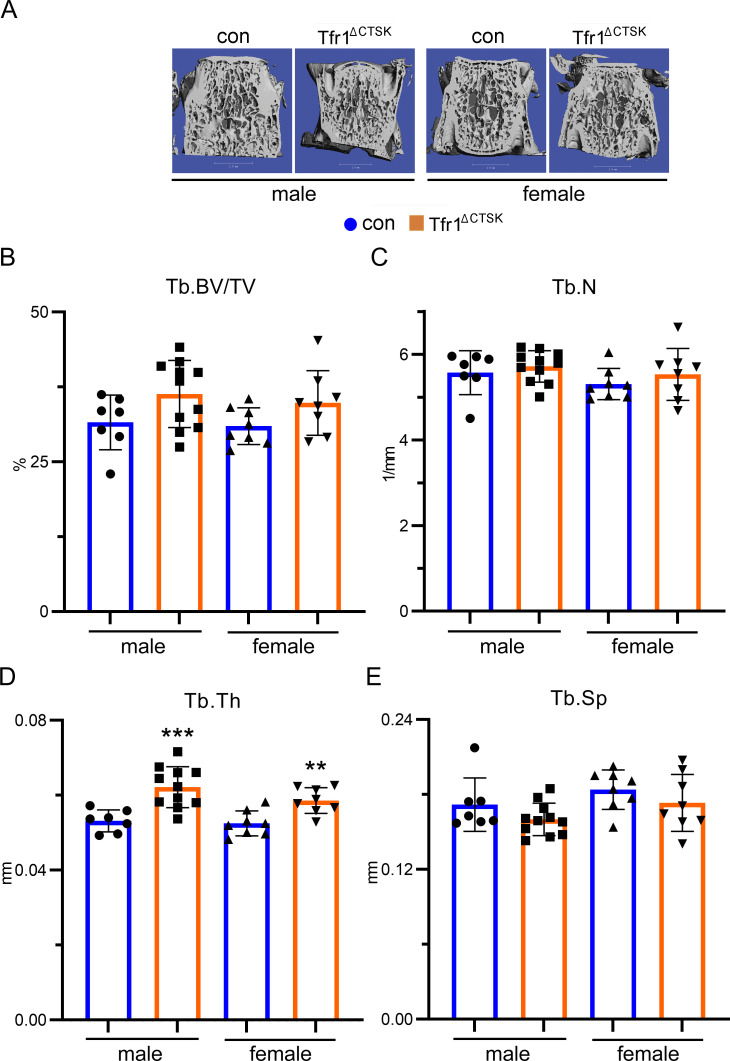
Deletion of Tfr1 in cathepsin K-Cre expressing osteoclasts slightly increases trabecular thickness of lumber vertebrae of 10-week-old male and female mice. (**A**) Representative µCT images of L4 lumber vertebra of male and female control (con) and conditional knockout (Tfr1^ΔCTSK^) mice on the 129×C57BL6J mixed background. scale bar = 1 mm. (**B–E**) µCT analysis of bone mass and structure of L4 lumber vertebra. Tb: trabecular bone; BV/TV: bone volume/tissue volume; Tb.N: trabecular number; Tb.Th: trabecular thickness; Tb.Sp: trabecular spacing. The data are presented as mean ± SD. n=7–11. ** p<0.01, *** p<0.001 vs con by one-way ANOVA. Figure 2—figure supplement 7—source data 1.Deletion of Tfr1 in cathepsin K-Cre expressing osteoclasts slightly increases trabecular thickness of lumber vertebrae of 10-week-old male and female mice.

**Figure 2—figure supplement 8.**
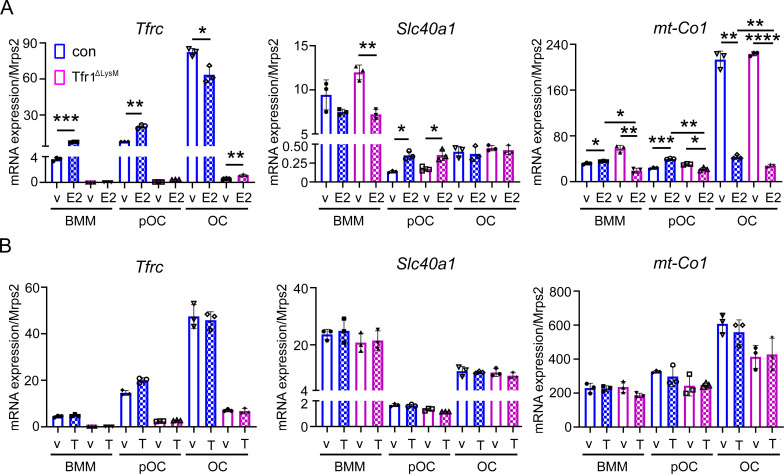
Estrogen but not androgen synergistically with Tfr1-deficiency inhibits the mRNA expression of mitochondrial cytochrome c oxidase subunit I in osteoclast lineage cells. Quantitative PCR detection of the mRNA levels of *Tfrc*, *Fpn*, and *mt-Co1* in vehicle (**v**), estrogen (**E2**), and androgen (**T**) treated control (con) and Tfr1^ΔLysM^ osteoclast lineage cells. The data are presented as mean ± SD. n=3. * p<0.05; ** p<0.01; *** p<0.001, **** p<0.0001 vs vehicle or control analyzed by one-way ANOVA. Figure 2—figure supplement 8—source data 1.Estrogen but not androgen synergistically with Tfr1-deficiency inhibits the mRNA expression of mitochondrial cytochrome c oxidase subunit I in osteoclast lineage cells.

**Figure 2—figure supplement 9.**
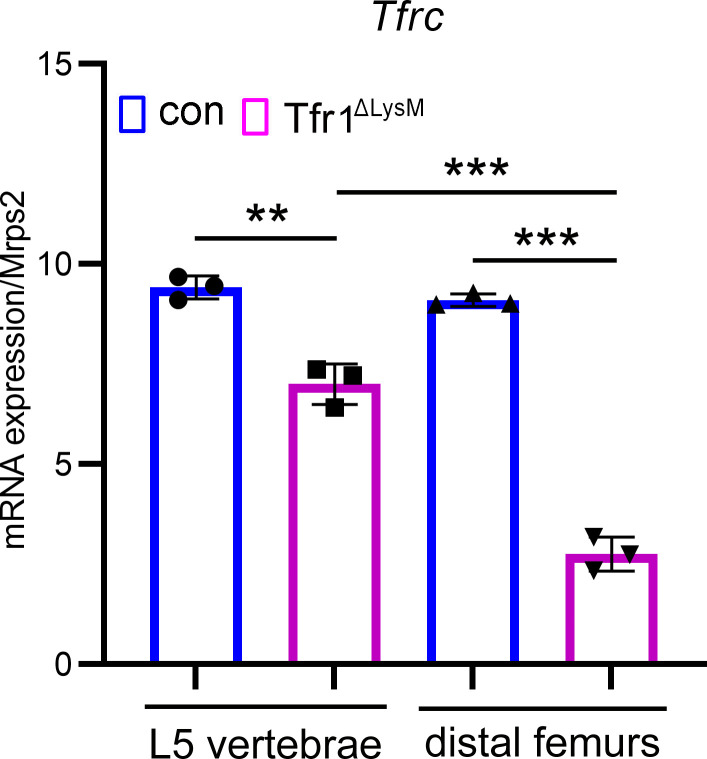
The deletion efficiency of Tfr1 by LysM-Cre is more potent in the distal femur than in the lumber vertebra as examined by quantitative PCR. The data are presented as mean ± SD. n=3. ** p<0.01 and *** p<0.001 vs control (con) or vertebra analyzed by one-way ANOVA. Figure 2—figure supplement 9—source data 1.The deletion efficiency of Tfr1 by LysM-Cre is more potent in the distal femur than in the lumber vertebra as examined by quantitative PCR.

**Figure 3.**
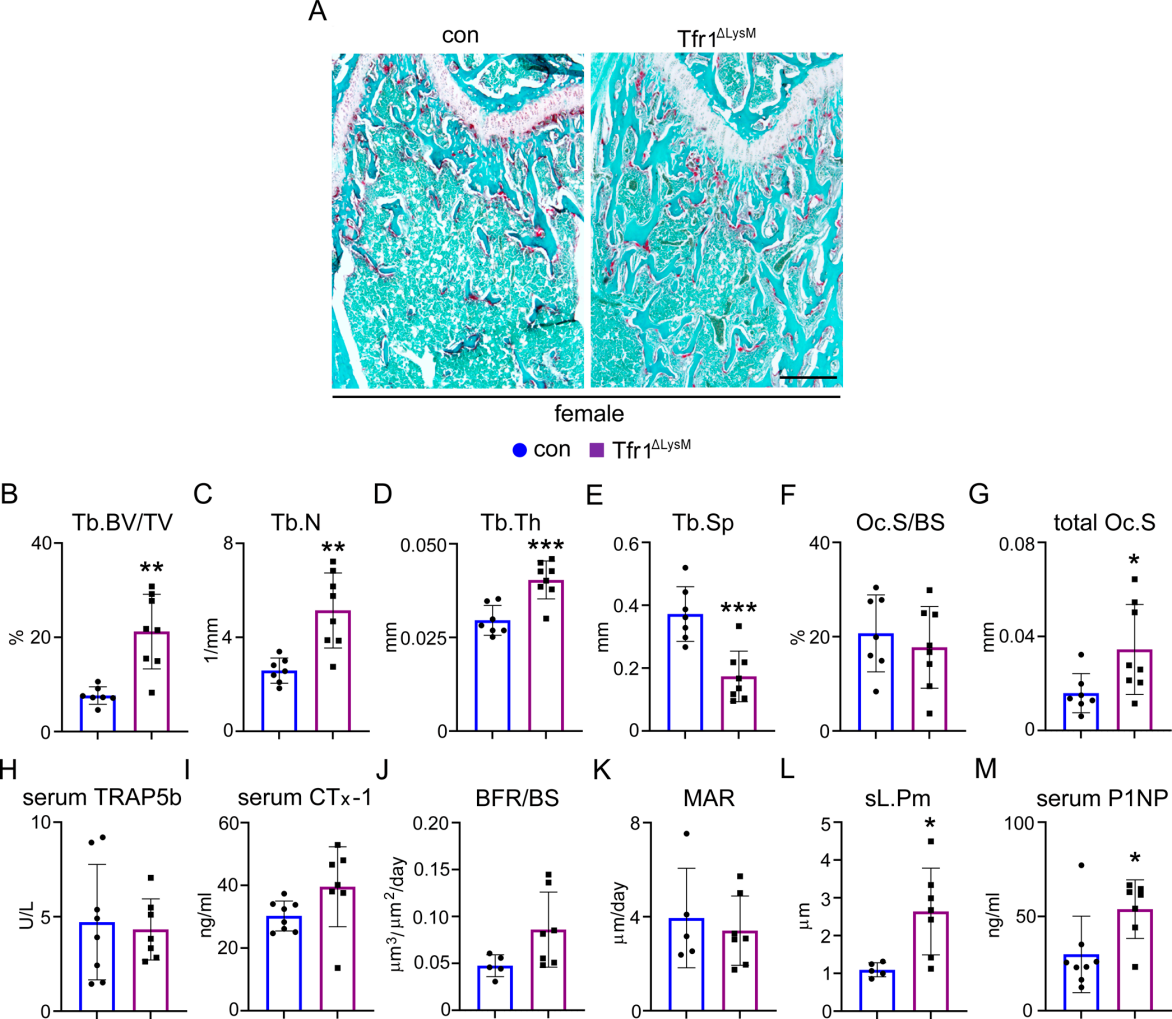
Loss of *Tfr1* in myeloid osteoclast precursors has no impacts on osteoclast number and bone formation in 10-week-old female mice. (**A**) Images of fast green and TRAP staining (scale bar = 200 μm) and (**B–G**) histomorphometric analysis of the metaphysis of decalcified distal femur histological sections of 10-week-old female control (con) and Tfr1^ΔLysM^ mice. (**H**), (**I**), and (**M**) quantitative measurements of serum markers for bone resorption and bone formation by ELISA. (**J–L**) Dynamic histomorphometry analysis of tetracycline-labeled sections from undecalcified distal femurs. Tb: trabecular bone; BV/TV: bone volume/tissue volume; Tb.N: trabecular number; Tb.Th: trabecular thickness; Tb.Sp: trabecular spacing; Oc.S/BS: osteoclast surface/bone surface; BFR: bone formation rate; MAR: mineral apposition rate; sL.Pm: single tetracycline labeled surface. The data are presented as mean ± SD. n=5–13. * p<0.05, ** p<0.01, and *** p<0.001 vs con by one-way ANOVA. Figure 3—source data 1.Loss of *Tfr1* in myeloid osteoclast precursors has no impacts on osteoclast number and bone formation in 10-week-old female mice.

To determine the cellular processes that contribute to increased trabecular bone mass caused by deletion of Tfr1 in myeloid cells in mice, we performed histology and histomorphometry analysis of distal femurs from 10-week-old male and female Tfr1^ΔlysM^ mice and their corresponding controls. Consistent with the µCT data, fast green and TRAP (tartrate-resistant acid phosphatase) staining of decalcified paraffin-embedded sections (Figure 3A and Figure 3—figure supplement 1A) demonstrated increased trabecular mass, Tb.N, Tb.Th, and decreased Tb.Sp in female but not male Tfr1^ΔlysM^ mice compared to their control littermates (Figure 3B-E and Figure 3—figure supplement 1B–1E). While the TRAP stained osteoclast surface adjusted by bone surface (Oc.S/BS), a histological parameter for osteoclast density, was the same in Tfr1^ΔlysM^ mice of both genders as in control mice (Figure 3F and Figure 3—figure supplement 1F), the total Oc.S which reflects the number of osteoclasts was increased in female Tfr1^ΔlysM^ but not male mice due to expanded trabecular bone mass in female knockout mice (Figure 3G and Figure 3—figure supplement 1G). The increased number of osteoclasts in distal femurs of Tfr1^ΔlysM^ female mice compared to control female mice was further supported by a >twofold higher level of mRNAs of osteoclast marker genes, *Acp5* (encoding TRAP) as measured by quantitative PCR (qPCR) (Figure 3—figure supplement 2). However, serum levels of bone resorption markers, TRAP5b and CTx-I, were similar between the control and Tfr1 deficient female mice (Figure 3H-I and Figure 3—figure supplement 1H–1I), thus reflecting a likely decrease in the activity of individual osteoclasts in female Tfr1^ΔlysM^ mice in vivo. Further experiments are needed to confirm this assertion.

**Figure 3—figure supplement 1.**
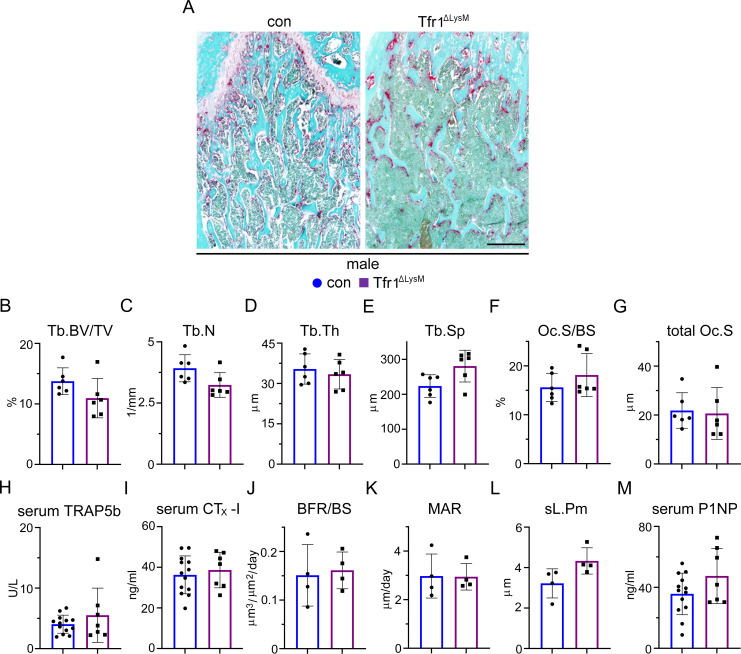
Loss of *Tfr1* in myeloid osteoclast precursors has no impacts on osteoclast number and bone formation in 10-week-old male mice. (**A**) Images of fast green and TRAP staining (scale bar = 200 μm) and (**B–G**) histomorphometric analysis of the metaphysis of decalcified distal femur histological sections of 10-week-old male control (con) and Tfr1^ΔLysM^ mice. (**H**), (**I**), and (**M**) quantitative measurements of serum markers for bone resorption and bone formation by ELISA. (**J–L**) Dynamic histomorphometry analysis of tetracycline-labeled sections from undecalcified distal femurs. Tb: trabecular bone; BV/TV: bone volume/tissue volume; Tb.N: trabecular number; Tb.Th: trabecular thickness; Tb.Sp: trabecular spacing; Oc.S/BS: osteoclast surface/bone surface; BFR: bone formation rate; MAR: mineral apposition rate; sL.Pm: single tetracycline labeled surface. The data are presented as mean ± SD and analyzed by Student’s t-test. n=4-13. Figure 3—figure supplement 1—source data 1.Loss of *Tfr1* in myeloid osteoclast precursors has no impacts on osteoclast number and bone formation in 10-week-old male mice.

**Figure 3—figure supplement 2.**
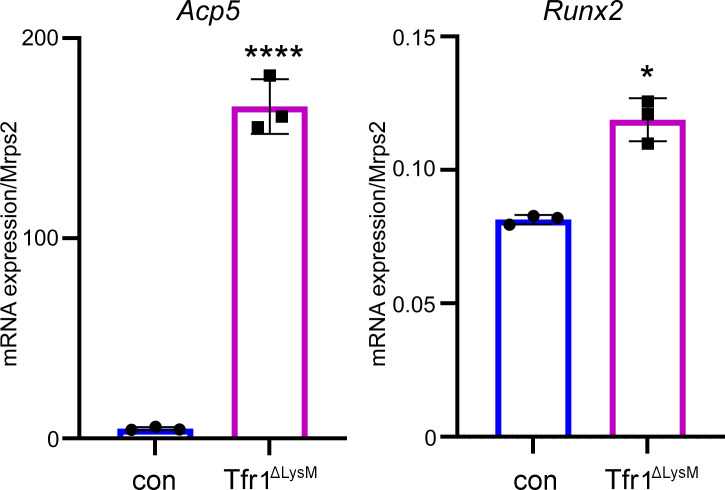
The mRNA levels of osteoclast marker gene *Acp5* and osteoblast marker gene *Runx2* increase in the distal femurs of female Tfr1^ΔLysM^ mice. Quantitative PCR detect of the mRNA expression of Acp5 and Runx2 in the distal femurs of control (con) and Tfr1^ΔLysM^ 10-week-old female mice. The data are presented as mean ± SD. n=3. * p<0.05 and **** p<0.0001 vs con analyzed by Student’s t-test. Figure 3—figure supplement 2—source data 1.The mRNA levels of osteoclast marker gene *Acp5* and osteoblast marker gene *Runx2* increase in the distal femurs of female Tfr1^ΔLysM^ mice.

**Figure 3—figure supplement 3.**
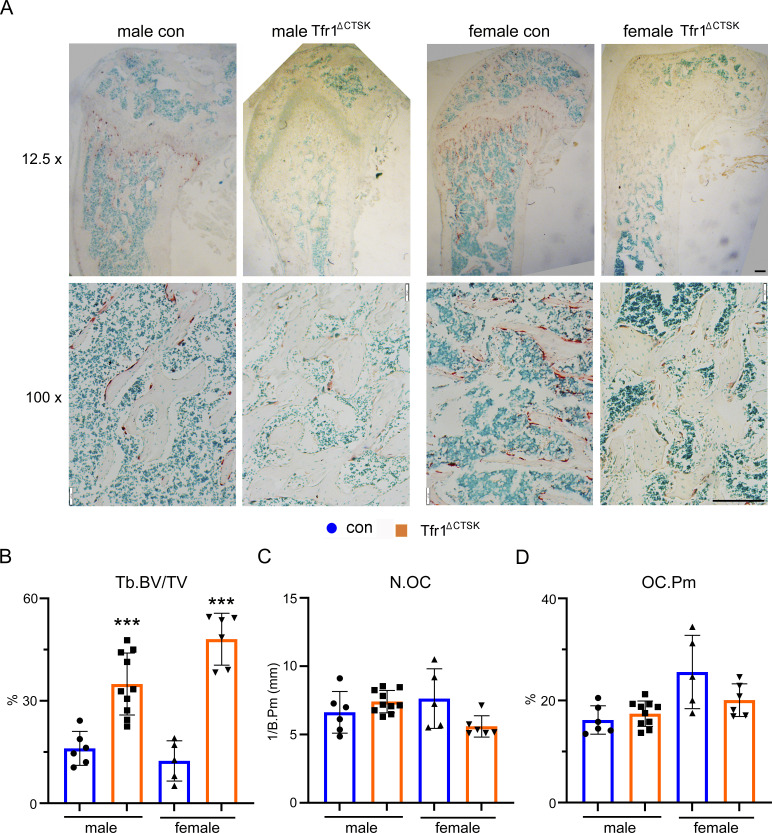
Deletion of *Tfr1* in cathepsin K-Cre expressing osteoclasts increases trabecular bone mass without influence on osteoclastogenesis in vivo. (**A**) The low (12.5×) and high (100×) magnification of images of TRAP staining of histological sections of decalcified distal femurs of 10-week-old control (con) and *Tfr1* conditional knockout (Tfr1^ΔCTSK^) mice on the 129×C57BL6J mixed background. scale bars = 20 μm and 80 μm, respectively. (**B–D**) Histomorphometric analysis of trabecular bone mass and osteoclast number. BV/TV: bone volume/tissue volume; N.OC: osteoclast number/mm bone surface; OC.Pm: percentage of osteoclast surface/bone surface. The data are presented as mean ± SD. n=5–10. *** p<0.001 vs control by one-way ANOVA. Figure 3—figure supplement 3—source data 1.Deletion of *Tfr1* in cathepsin K-Cre expressing osteoclasts increases trabecular bone mass without influence on osteoclastogenesis in vivo.

Since osteoclast-mediated bone resorption and osteoblast-mediated bone formation are coupled and regulated from each other, we also examined bone formation activities of osteoblasts in control and Tfr1^ΔlysM^ mice by dynamic bone histomorphometry analysis of tetracycline-labeled plastic-embedded tissue sections. As shown in Figure 3J-K and Figure 3—figure supplement 1J–1K, bone formation rate and osteoblast mineral apposition rate in trabecular bone were similar between control and Tfr1^ΔlysM^ male and female mice. The single tetracycline-labeled surface (sL.Pm) in female but not male Tfr1^ΔlysM^ mice was higher than their control mice, likely due to increased trabecular bone mass/surface (Figure 3L and Figure 3—figure supplement 1L). The increased serum level of P1NP (Figure 3M and Figure 3—figure supplement 1M), a systemic bone formation marker, in female but not male Tfr1^ΔlysM^ mice may be caused by increased trabecular bone and total number of osteoblasts in these mice. Accordingly, expression level osteoblast marker gene *Runx2* was increased in the distal femurs of Tfr1^ΔlysM^ female mice relative to their controls (Figure 3—figure supplement 2). Based on what is known about osteoclast-mediated coupling of bone formation, one would predict that the reduced osteoclast activity in Tfr1^ΔLysM^ mice should reduce bone formation. By contrast, the increased serum PINP (Procollagen 1 N-Terminal Propeptide) levels and Runx2 expression in these mice suggest increased bone formation. Thus, we do not have an explanation at present if the increased bone formation is a direct consequence of inhibition of osteoclast activity or by some other mechanisms. Further studies are needed to address this issue.

### Deletion of Tfr1 in cathepsin K-Cre expressing differentiated osteoclasts increases trabecular bone mass of long bones in both male and female mice

To determine the role of Tfr1 in differentiated osteoclasts during bone remodeling, we crossed *Tfrc*-flox mice with *Ctsk*-Cre knock-in mice, a Cre-driver that has been extensively used to disrupt genes of interest in differentiated osteoclasts (Fujiwara et al., 2016; Wang et al., 2018; Nakamura et al., 2007). The progeny of *Tfrc*^+/+^;*Ctsk*^Cre/+^ and *Tfrc*^flox/flox^;Ctsk^Cre/+^ congenic mice on a C57BL6/129 mixed background was used as control and osteoclast conditional knockout (Tfr1^ΔCTSK^) mice, respectively. Both male and female Tfr1^ΔCTSK^ mice were born and developed normally. The body size and weight of 10-week-old male and female Tfr1^ΔCTSK^ mice were similar to their littermate gender matched control mice (data not shown). The µCT examination of distal femurs uncovered a more than twofold increase of trabecular BV/TV with increased Tb.N, thickness, and decreased Tb.Sp in both male and female Tfr1^ΔCTSK^ mice compared to their littermate controls (Figure 2—figure supplement 6A–6E). A slight increase in Cort.Th was only observed in female Tfr1^ΔCTSK^ mice (Figure 2—figure supplement 6F). Similar to Tfr1^ΔlysM^ mice, loss of Tfr1 in differentiated osteoclasts had little effects on trabecular bone mass and structure except an increase in Tb.Th in vertebral bones of male and female Tfr1^ΔCTSK^ mice (Figure 2—figure supplement 7).

Histology and histomorphometry analysis of paraffin-embedded, TRAP-stained femoral sections demonstrated dramatic increase in trabecular BV/TV in both male and female Tfr1^ΔCTSK^ mice relative to their control mice (Figure 3—figure supplement 3A and B). These data are consistent with the µCT results shown in Figure 2—figure supplement 6. The number of TRAP-positive osteoclasts per bone perimeter as well as the percentage of osteoclast-covered bone surface in Tfr1^ΔCTSK^ mice of either genders were not different from their respective control mice (Figure 3—figure supplement 3C–3D). These results indicate that loss of Tfr1 in *Ctsk*-Cre expressing osteoclasts increases trabecular bone mass but has no effects on osteoclastogenesis in vivo.

### Tfr1-mediated iron uptake plays an important role in mature osteoclast actin cytoskeletal organization and bone resorption in vitro

**Figure 4.**
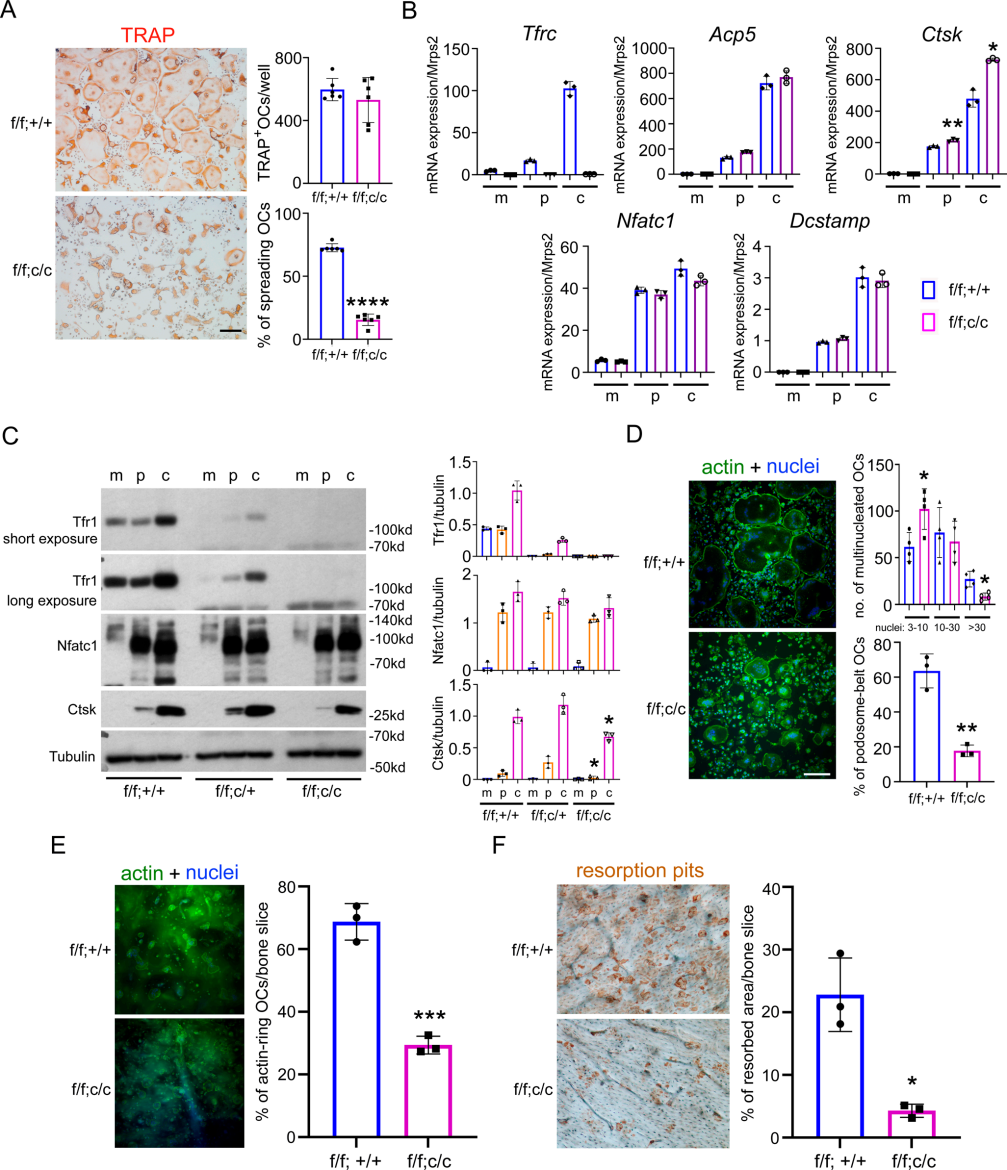
Deletion of Tfr1 in osteoclast myeloid precursors has no effects on osteoclast differentiation but attenuates cytoskeleton organization and bone resorption in mature osteoclasts cultured from female mice. (**A**) TRAP staining (scale bar = 10 μm) and quantification of numbers of total and spreading osteoclasts (OCs) cultured on plastic 24-well dishes. n=6. (**B**) Quantitative PCR detection of the mRNA expression of Tfr1 and osteoclast marker genes relative to the mitochondrial gene *Mrps2. Tfrc*, encoding Tfr1; *Acp5*, encoding TRAP; *Ctsk*, encoding Cathepsin K; *Nfatc1*, encoding NFATc1; *Dcstamp*, encoding DC-Stamp. n=3. (**C**) The protein level of Tfr1 and osteoclast markers, NFATc1 and Cathepsin K (Ctsk), in bone marrow monocytes (**m**), mono-nuclear pre-osteoclasts (**p**), and mature osteoclasts (**c**) was detected by western blotting and quantified by densitometry using the NIH ImageJ software. Tubulin served as loading control. n=3. (**D**) The actin filaments and nuclear were stained by Alexa-488 conjugated Phalloidin and Hoechst 33258, respectively, in osteoclasts cultured on glass coverslips (scale bar = 10 μm). The number of osteoclasts with different nuclei and the percentage of spreading osteoclasts with the peripheral distributed podosome-belt were counted. n=3–4. (**E**) The actin filaments and nuclear were stained by Alexa-488 conjugated Phalloidin and Hoechst 33342, respectively, in osteoclasts cultured on cortical bovine bone slices (scale bar = 10 μm). The total number of osteoclasts and the number of osteoclasts with actin-ring were counted, and the percentage of active osteoclasts per bone slices was calculated. n=3. (**F**) Resorption pits were stained by horseradish peroxidase conjugated wheat-germ agglutinin (scale bar = 20 μm). The percentage of resorbed area per bone slice was calculated using the NIH ImageJ software. The data are presented as mean ± SD. n=3. * p<0.05; ** p<0.01; *** p<0.001;**** p<0.0001 vs control (f/f;+/+) by one-way ANOVA and Student’s t-test. Figure 4—source data 1.Deletion of Tfr1 in osteoclast myeloid precursors has no effects on osteoclast differentiation but attenuates cytoskeleton organization and bone resorption in mature osteoclasts cultured from female mice. Figure 4—source data 2.Immunoblotting for Figure 4C.

To further identify the cellular and molecular mechanisms by which Tfr1 and Tfr1-mediated iron uptake regulate osteoclasts, we isolated bone marrow cells from female and male control and Tfr1^ΔlysM^ mice and cultured them with M-CSF plus RANKL for 4 days to induce osteoclast differentiation in vitro. The total number of TRAP^+^ multinucleated osteoclasts (with ≥3 nuclei per cell) in Tfr1^ΔlysM^ cultures of both genders was not different from their respective controls. However, the Tfr1-deficient female and male osteoclasts displayed a dramatic defect in mature osteoclast spreading (Figure 4 and Figure 4—figure supplement 1A). To further corroborate the effects of Tfr1-deletion on osteoclast differentiation, we measured the mRNA expression of the osteoclast marker genes *Acp5*, *Ctsk*, the master transcription factor of osteoclast differentiation *Nfatc1*, and the osteoclast fusion regulator *Dcstamp* (encoding DC-Stamp) in female control and Tfr1^ΔlysM^ BMM, mononuclear pre-osteoclasts, and multinucleated mature osteoclasts by qPCR. As shown in Figure 4B, the mRNA levels of *Acp5*, *Nfatc1*, and *Dcstamp* in Tfr1^ΔlysM^ in osteoclast precursors as well as mature osteoclasts were similar to those of respective control cells. The mRNA expression of Ctsk in Tfr1^ΔlysM^ pre-osteoclasts and mature osteoclasts was slightly higher than in control cells.

**Figure 4—figure supplement 1.**
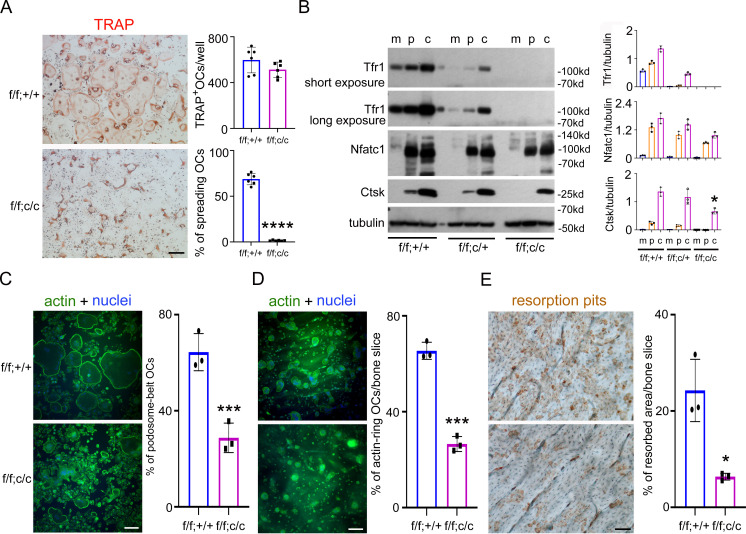
Deletion of Tfr1 in osteoclast myeloid precursors has no effects on osteoclast differentiation but attenuates cytoskeleton organization and bone resorption in mature osteoclasts cultured from male mice. (**A**) TRAP staining (scale bar = 10 μm) and quantification of numbers of total and spreading osteoclasts (OCs) cultured on plastic 24-well dishes. n=6. (**B**) The protein level of Tfr1 and osteoclast markers, NFATc1 and Cathepsin K (Ctsk), in bone marrow monocytes (**m**), mono-nuclear pre-osteoclasts (**p**), and mature osteoclasts (**c**) was detected by western blotting and quantified by densitometry using the NIH ImageJ software. Tubulin served as loading control. n=3. (**C**) The actin filaments and nuclear were stained by Alexa-488 conjugated Phalloidin and Hoechst 33258, respectively, in osteoclasts cultured on glass coverslips (scale bar = 10 μm). The percentage of spreading osteoclasts with the peripheral distributed podosome-belt were counted. n=3–4. (**D**) The actin filaments and nuclear were stained by Alexa-488 conjugated Phalloidin and Hoechst 33342, respectively, in osteoclasts cultured on cortical bovine bone slices (scale bar = 10 μm). The total number of osteoclasts and the number of osteoclasts with actin-ring were counted and the percentage of active osteoclasts per bone slices was calculated. n=3. (**E**) Resorption pits were stained by horseradish peroxidase conjugated wheat-germ agglutinin (scale bar = 20 μm). The percentage of resorbed area per bone slice was calculated using the NIH ImageJ software. The data are presented as mean ± SD. n=3. * p<0.05, *** p<0.001, and **** p<0.0001 vs control (f/f;+/+) by one-way ANOVA and Student’s t-test. Figure 4—figure supplement 1—source data 1.Deletion of Tfr1 in osteoclast myeloid precursors has no effects on osteoclast differentiation but attenuates cytoskeleton organization and bone resorption in mature osteoclasts cultured from male mice. Figure 4—figure supplement 1—source data 2.Immunoblotting for Figure 4—figure supplement 1B.

Next, we examined the protein levels of Tfr1, Nfatc1, and Ctsk in control and Tfr1-deficient osteoclast lineage cells by immunoblotting and quantified the specific bands of these osteoclast markers by densitometry using NIH Image J software. As demonstrated in Figure 4C and Figure 4—figure supplement 1B, deletion of Tfr1 by two copies of *Lyz2*-Cre (f/f;c/c) in both female and male osteoclast lineage cells had no effects on the protein level of Nfatc1 in osteoclast precursor and mature cells, whereas the level of CtsK in Tfr1^ΔlysM^ pre- and mature-osteoclasts was slightly decreased compared to control (f/f;+/+) and Tfr1 heterozygous (f/f;c/+) osteoclasts. The decreased Ctsk protein level in Tfr1^ΔlysM^ osteoclasts might contribute to impaired bone resorption in these cells as shown in Figure 4F. To characterize at which stage the Tfr1-deletion affects Ctsk expression and osteoclastogenesis, we counted the number of osteoclasts with 3–10, 10–30, and >30 nuclei cultured from female mice, respectively. As shown in the upper-right panel of Figure 4D, the number of osteoclasts with 3–10 nuclei in Tfr1^ΔlysM^ culture was higher than control. While there was no difference in the number of osteoclasts with 10–30 nuclei between control and Tfr1^ΔlysM^ cultures, the number of large osteoclasts with >30 nuclei in Tfr1^ΔlysM^ culture was lower than control. However, the mRNA expression of osteoclast fusion gene, *Dcstamp,* was similar in control and Tfr1^ΔlysM^ osteoclasts. The exact mechanism(s) of decreased fusion in the late stage of osteoclastogenesis in Tfr1^ΔlysM^ cultures need further investigation in future.

Since actin cytoskeleton organization plays an essential role in osteoclast spreading and the formation of podosome-belts in osteoclasts cultured on plastic dishes and actin-rings in osteoclasts cultured on bone matrix, we then stained actin filaments with Alexa-488 conjugated Phalloidin in control and Tfr1-deficient female and male osteoclasts cultured on plastic and bone. While the podosome-belts and actin-rings formed normally in control osteoclasts, these actin-based structures were dysregulated in Tfr1-deficient female and male osteoclasts (Figure 4D-E and Figure 4—figure supplement 1C–1D). Since formation of actin-rings is a hallmark of osteoclast activation and function, these results indicate that loss of Tfr1 in osteoclast suppresses osteoclast function. Supporting this hypothesis, we found that Tfr1-deficient osteoclasts resorbed less bone than controls as revealed by the resorption-pit staining on cortical bovine bone slices (Figure 4F and Figure 4—figure supplement 1E). Tfr1 ^ΔCtsk^ osteoclasts displayed similar in vitro phenotype as Tfr1^ΔlysM^ osteoclasts (data not shown).

**Figure 5.**
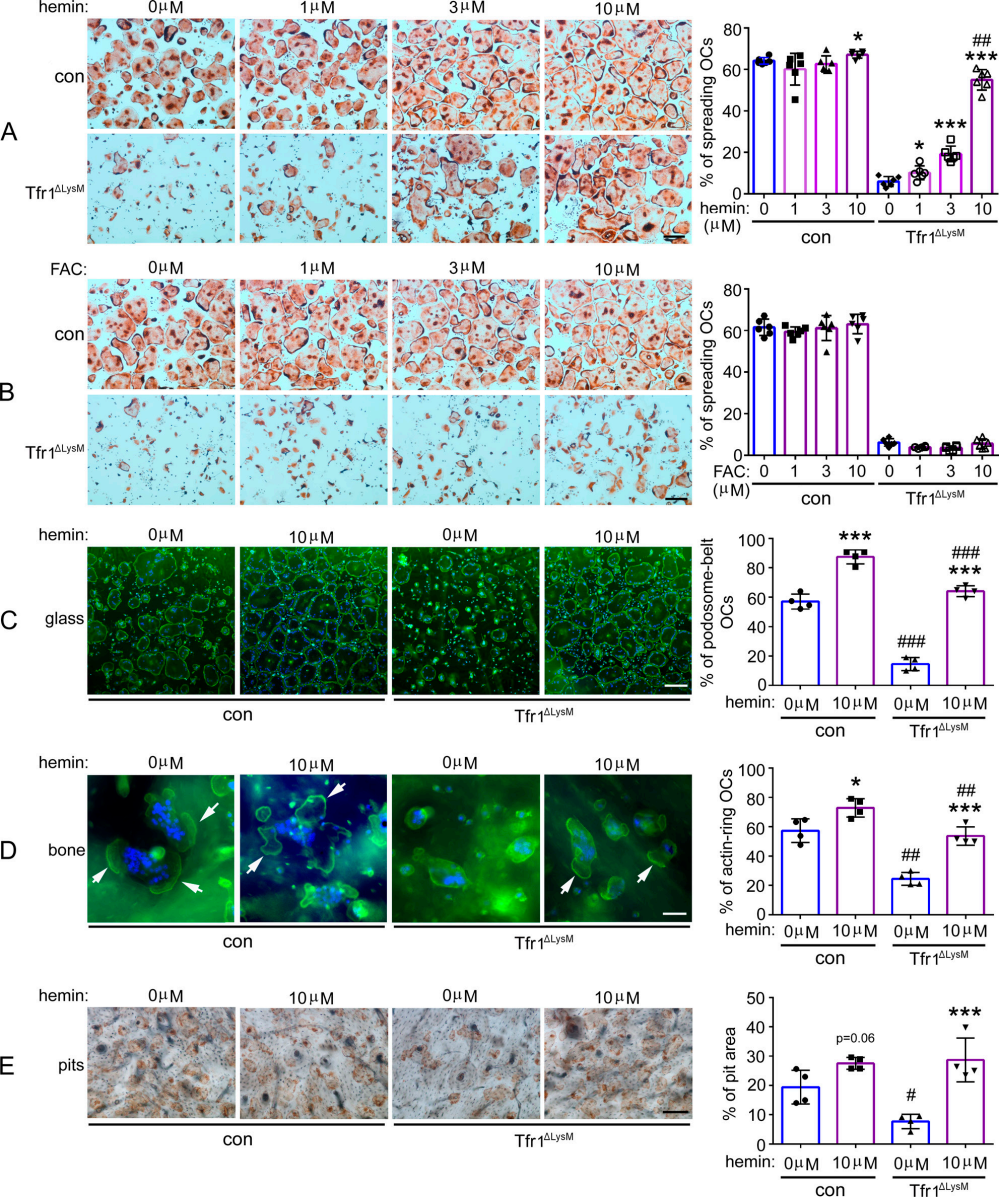
High dose of hemin but not ferric ammonium citrate rescues the phenotypes of Tfr1-deficient osteoclasts. (**A**) and (**B**) TRAP staining and quantification of the number of spreading osteoclasts in control (con) and Tfr1^ΔLysM^ osteoclast cultures. n=6. scale bar = 10 μm. (**C**) and (**D**) Staining of actin filaments and nuclear and quantification of the number of podosome-belt and actin-ring osteoclasts cultured on glass coverslips and bone slices, respectively. n=4. scale bar = 10 μm. (**E**) Resorption pit staining in con and Tfr1^ΔLysM^ cultures. Scale bar = 20 μm. The data are presented as mean ± SD. n=4. * p<0.05 and *** p<0.001 vs vehicle-treated cells; # p<0.05, ## p<0.01, and ### p<0.001 vs vehicle- or 10 μM hemin-treated control cells, respectively, by one-way ANOVA. Figure 5—source data 1.High dose of hemin but not ferric ammonium citrate rescues the phenotypes of Tfr1-deficient osteoclasts.

Besides regulating iron-uptake, Tfr1 also plays non-canonical roles in regulation of intestinal epithelial homeostasis and mitochondrial morphology and function (Chen et al., 2015; Senyilmaz et al., 2015). To determine whether the defects in cytoskeleton organization and resorptive function of Tfr1-deficient osteoclasts were caused by low intracellular iron content, we treated control and Tfr1^ΔlysM^ osteoclast cultures with increased doses of hemin (ferric chloride heme) and ferric ammonium citrate (FAC), which are transported into cells via heme transporters Hcp1 (encoded by *Slc46a1*) and NTBI transporter Zip14 (encoded by *Slc39a14*), respectively (Figure 1—figure supplement 1). While 10 µM of hemin slightly stimulated osteoclast spreading, cytoskeleton organization, and bone resorption in control osteoclasts, hemin dose-dependently increased the number of spreading osteoclasts in Tfr1-deficient osteoclasts (Figure 5A). In contrast, FAC had no effects on control or Tfr1-deficient osteoclasts (Figure 5B). At 10 µM concentration, hemin significantly rescued the cytoskeleton organization and bone resorption defects of Tfr1-depleted osteoclasts (Figure 5C-E). These results also suggest that heme-uptake, but not NTBI and ferritin pathways, is an alternative iron acquiring route in osteoclasts in the absence of Tfr1. Therefore, both our in vivo and in vitro findings unveil a critical role of Tfr1-mediated cellular iron homeostasis in osteoclast activation/function. It should be noted that even high concentration (10 μM) of hemin can only partially rescue the defects in osteoclast spreading and cytoskeleton organization (Figure 5A, C and D), indicating that osteoclasts have limited alternative iron acquisition pathways in the absence of Tfr1 as in other cells.

### Depletion of Tfr1 in osteoclast lineage cells attenuates mitochondrial biogenesis, ROS production, and OXPHOS predominantly in mature osteoclasts

**Figure 6.**
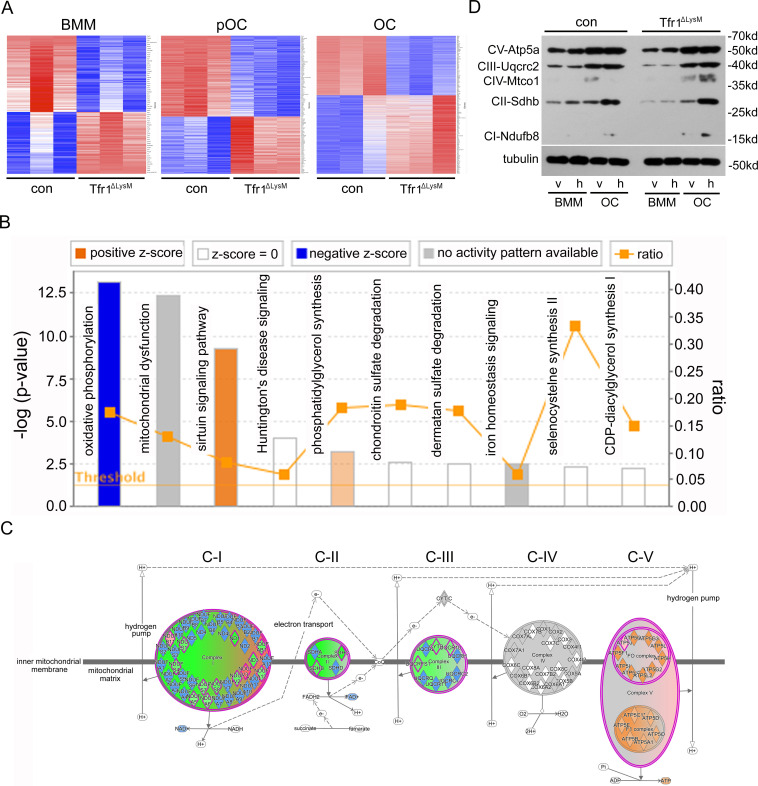
The mitochondrial oxidative phosphorylation is mostly affected by Tfr1-deficiency in mature osteoclasts. (**A**) Heatmaps of proteins that are differentially regulated in control (con) and Tfr1^ΔLysM^ bone marrow monocytes (BMM), mononuclear pre-osteoclasts (pOC), and mature osteoclasts (OC) identified by quantitative proteomics. (**B**) The signaling pathways that are affected by Tfr1-deficiency in mature osteoclasts identified by Ingenuity pathway analysis (IPA). (**C**) The changes of proteins along the mitochondrial respiration chain that are regulated by Tfr1 in mature osteoclasts. C-I to C-V: mitochondrial respiratory complex-I to complex-V. (**D**) Western blotting detection of the components of mitochondrial respiratory C-I to C-V in vehicle and 10 μM hemin treated con and Tfr1^ΔLysM^ BMM and OCs. Figure 6—source data 1.Immunoblotting for Figure 6D.

To determine which intracellular pathways are most affected by loss of Tfr1 in osteoclast lineage cells, we did quantitative proteomic analysis to identify proteins that are up- or downregulated in Tfr1-deficient BMM, mononuclear osteoclast precursors, and multinucleated mature osteoclasts. The heatmaps were generated using the ‘heatmap’ function in the R-package (Figure 6A). By a 1.2-log2fold cut-off, we found that a total of 135, 497, and 2562 proteins were influenced by loss of Tfr1 in monocytes, pre-osteoclasts, and mature osteoclasts, respectively (Supplementary files 1–3). The abundance of proteins involved in Tf-dependent iron uptake pathway, including Tfr1, Steap3/4, and DMT1, was all decreased in Tfr1-deficient cells (Supplementary files 1–3). The greater number of affected proteins in mature osteoclasts correlated with a high level of Tfr1 and Tfr1-mediated iron uptake in osteoclasts (Figure 1). By Ingenuity pathway analysis (IPA) analysis, we identified that the mitochondrial OXPHOS pathway and sirtuin signaling pathway were the most significantly down- and upregulated pathways in mature osteoclasts, respectively (Figure 6B and Supplementary file 4). Among the mitochondrial proteins affected by loss of Tfr1 and Tf-dependent iron uptake in osteoclasts, the levels of proteins functioning in mitochondrial respiratory complex I to complex III decreased, whereas the proteins participating in complex V, that lack heme and Fe-S clusters (Figure 1—figure supplement 1), were increased probably by a compensate mechanism. The proteins in the complex IV were not affected by iron-deficiency (Figure 6C and Supplementary file 4). To consolidate the finding from quantitative proteomic results that cellular iron regulates the components of mitochondrial respiration chain, we did immunoblotting to detect the changes of mitochondrial respiratory complexes in control and Tfr1^ΔlysM^ monocytes and mature osteoclasts treated with either vehicle or 10 μM hemin using a cocktail of antibodies recognizing the representative components of mitochondrial respiratory complexes I–V. As shown in Figure 6D and Figure 6—figure supplement 1, there was an obvious decrease in the mitochondrial complexes II and III in Tfr1^ΔlysM^ monocytes and osteoclasts compared to corresponding control cells. Hemin stimulated the mitochondrial complexes I and II in control and complexes I to IV in Tfr1^ΔlysM^ osteoclasts, indicating that Tfr1-mediated iron uptake plays an important role in osteoclast mitochondrial OXPHOS pathway. Although Figure 6D provides complementary evidence for proteomic data shown in Figure 6B, C, it should be pointed out that the antibody cocktail used in this immunoblotting contains antibodies against only one component of each of five mitochondrial respiratory complexes. Thus, the result of Figure 6D could not reflect the complete changes of mitochondria in Tfr1-deficient osteoclast lineage cells.

**Figure 6—figure supplement 1.**
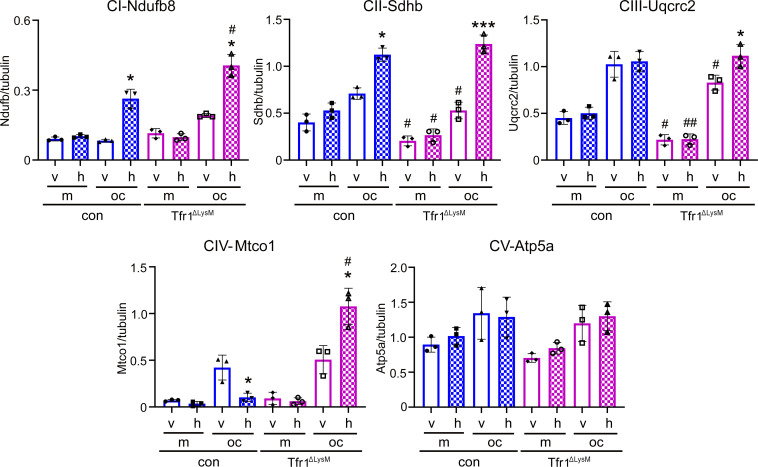
Quantification of immunoblotting bands in Figure 6D. Each band in immunoblots was quantified by densitometry using the NIH ImageJ software. n=3 from three immunoblots. * p<0.05 and *** p<0.001 vs the corresponding vehicle-treated cells of the same genotype; The data are presented as mean ± SD. # p<0.05 and ## p<0.01 vs the respective control cells of the same treatment by one-way ANOVA. Figure 6—figure supplement 1—source data 1.Quantification of immunoblotting bands in Figure 6D.

**Figure 7.**
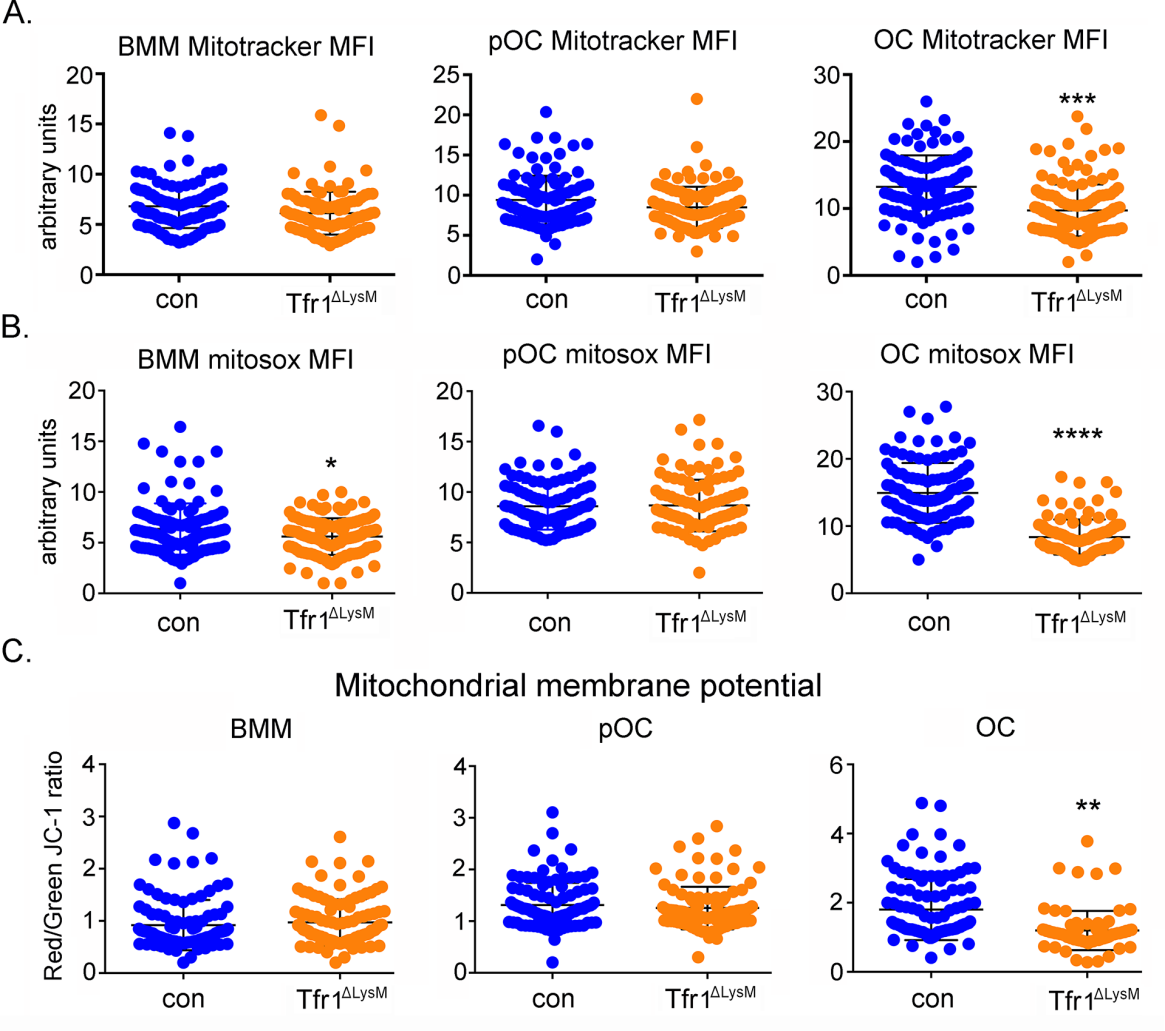
Loss of Tfr1 inhibits mitochondrial mass, reactive oxygen species (ROS) production, and membrane potential in mature osteoclasts. (**A**) Quantification of mitochondrial mass by Mito Tracker green staining in control (con) and Tfr1^ΔLysM^ bone marrow monocytes (BMM), mononuclear pre-osteoclasts (pOC), and mature osteoclasts (OC). (**B**) Measurement of mitochondria-derived ROS by Mitosox staining in con and Tfr1-deficient osteoclast lineage cells. (**C**) Measurement of mitochondrial membrane potential by JC-1 cationic carbocyanine dye staining in con and cKO osteoclast lineage cells. The data are presented as mean ± SD. n=25–50. ** p<0.01, *** p<0.001, and **** p<0.0001 vs con by unpaired Student’s t-test. MFI: mean fluorescent intensity per cell in arbitrary units. Figure 7—source data 1.Loss of Tfr1 inhibits mitochondrial mass, reactive oxygen species (ROS) production, and membrane potential in mature osteoclasts.

To further characterize how iron-deficiency modulates mitochondrial biogenesis and metabolism in osteoclast lineage cells, we first stained the control and Tfr1^ΔlysM^ osteoclast lineage cells with fluorescence-labeled Mito Tracker. Manual quantification of Mito Tracker fluorescent intensity of individual cells showed a small decrease in mitochondrial mass in Tfr1-deficient mature osteoclasts but not in monocytes and pre-osteoclasts (Figure 7A). Next, we examined the mitochondrion-derived ROS by MitoSOX-staining of control and Tfr1^ΔlysM^ cultured cells. As demonstrated in Figure 7B, loss of Tfr1 significantly decreased mitochondrial ROS production in monocytes and osteoclasts which was more pronounced in mature OCs. Insufficient iron in Tfr1^ΔlysM^ mature osteoclasts also resulted in a mild decrease in mitochondrial membrane potential (Figure 7C).

**Figure 8.**
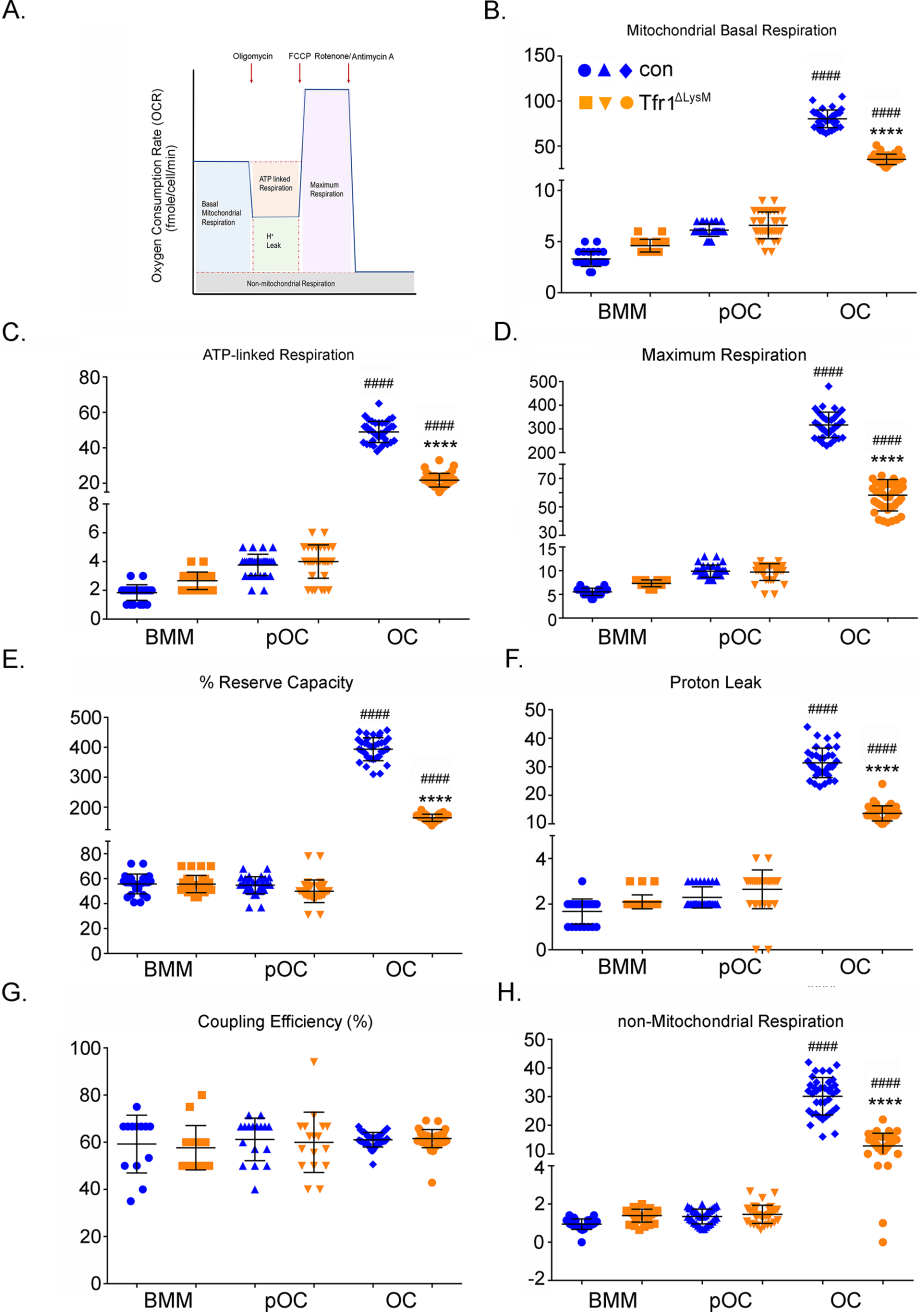
Tfr1-deletion in osteoclast lineage cells impairs mitochondrial and non-mitochondrial respirations in mature osteoclasts. (**A**) A graphic illustration of oxygen consumption measured by a Seahorse Extracellular Flux analyzer. FCCP: carbonyl cyanide p-trifluoro-methoxyphenyl hydrazone, a synthetic mitochondrial uncoupler. (**B–H**) Different fractions of mitochondrial and non-mitochondrial respirations per cell of control (blue) and Tfr1^ΔLysM^ (orange) bone marrow monocytes (BMM), mononuclear pre-osteoclasts (pOC), and mature osteoclasts (OC). The data are presented as mean ± SD. n=15-30. #### p<0.0001 vs BMM and pOC; **** p<0.0001 vs control OC by unpaired Student’s t-test. Figure 8—source data 1.Tfr1-deletion in osteoclast lineage cells impairs mitochondrial and non-mitochondrial respirations in mature osteoclasts.

Lastly, we measured mitochondrial respiration using the Seahorse Extracellular Flux Analyzer in control and Tfr1-deficient osteoclast lineage cells isolated. As depicted in Figure 8A, the basal mitochondrial respiration is the direct measure of oxygen consumption rate (OCR) attributed to the mitochondrial electron transport chain (ETC). The maximum respiration is induced by incubating cells with the uncoupler, carbonyl cyanide 4-(trifluoromethoxy)phenylhydrazone (FCCP). The ATP-linked respiration is measured by exposing cells to oligomycin, an inhibitor of ATP synthase complex V. The non-mitochondrial respiration, which is the remaining OCR when the ETC activity is completely abolished by a mixture of rotenone/antimycin A. This parameter reflects the portion of cellular respiration by oxygen-consuming enzymes such as NADPH (nicotinamide adenine dinucleotide phosphate) oxidases, heme oxygenases, and/or lipoxygenases. The residual OCR after inhibition by oligomycin is the measure of the protons pumped through ETC, which consumes oxygen without generating any ATP (due to inhibited ATP synthase activity by the drug) and is referred to the H^+^ leak. As shown in Figure 8B–H, all these mitochondrial respiratory parameters increased dramatically in control and TfR1-null mature osteoclasts compared to their respective precursors. However, there were >twofold decreases of these measures in Tfr1-deficient mature osteoclasts relative to control osteoclasts. Furthermore, we calculated the reserve respiratory capacity, which is the difference between the maximum and basal respiration that can be utilized in the event of a sudden energy demand in cells. We reported this as a percentage for each group of cells by using the formula of ([maximum OCR - basal OCR]/maximum OCR ×100). Again, the reserve respiratory capacity in Tfr1-null osteoclasts was greatly reduced when compared to control cells (Figure 8E). By contrast, there was no difference in the coupling efficiency (the ratio of ATP-linked OCR to basal OCR) of control and Tfr1^ΔlysM^ osteoclast lineage cells (Figure 8G).

### TfR1-mediated iron uptake modulates osteoclast actin cytoskeleton through the WAVE regulatory complex (WRC)

**Figure 9.**
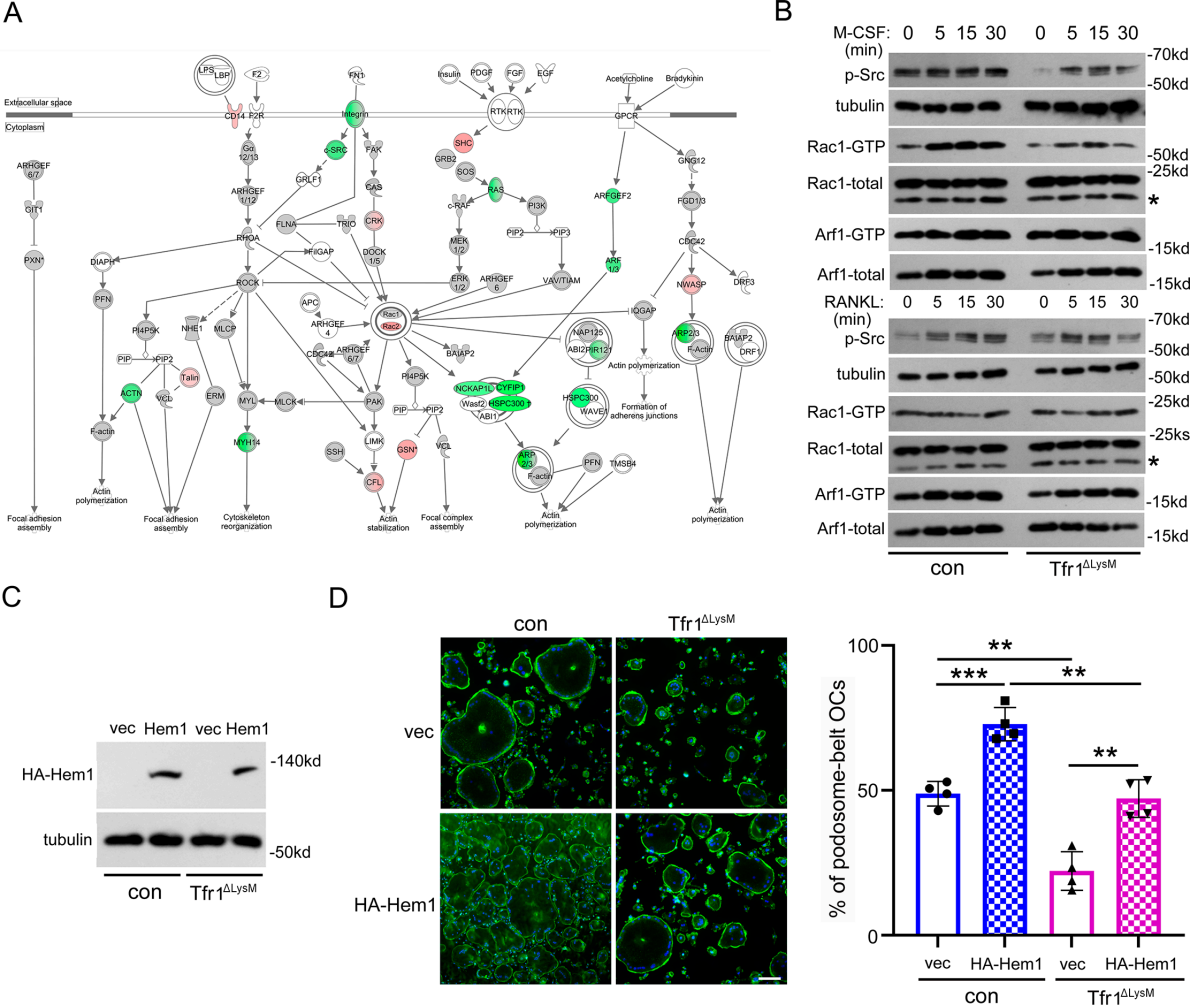
Loss of Tfr1 in osteoclasts inhibits the macrophage colony-stimulating factor (M-CSF) stimulated activation of c-Src and Rac1, and overexpression of Hem1 partially recuses the cytoskeletal organization defect in Tfr1-null osteoclasts. (**A**) A schematic map of cytoskeletal pathways generated by Ingenuity pathway analysis (IPA). The downregulated proteins are shown in green and upregulated proteins are marked in red orange. (**B**) Western blotting detection of M-CSF and receptor activator of NF-κB ligand (RANKL) induced phosphorylation of c-Src and the GTP-bound (active) form of small GTPase Rac1 and Arf1 in con and Tfr1^ΔLysM^ osteoclasts. The stars indicate unspecific bands. (**C**) Detection of HA-tagged Hem1 (encoded by Nckap1l) by western blotting in retroviral transduced con and Tfr1^ΔLysM^ bone marrow monocytes expressing empty vector (vec) and recombinant Hem1. Tubulin served as loading control. (**D**) The staining of actin filaments and nuclear in osteoclasts cultured on glass coverslips and quantification of the number of osteoclasts with podosome-belt. scale bar = 10 μm. The data are presented as mean ± SD. n=4. ** p<0.01 and *** p<0.001 by one-way ANOVA. Figure 9—source data 1.Loss of Tfr1 in osteoclasts inhibits the macrophage colony-stimulating factor (M-CSF) stimulated activation of c-Src and Rac1 and overexpression of Hem1 partially recuses the cytoskeletal organization defect in Tfr1-null osteoclasts. Figure 9—source data 2.Immunoblotting for Figure 9B.

To identify the mechanisms by which cellular iron and energy metabolism regulate osteoclast cytoskeleton, we turned to analyze the level of key osteoclast cytoskeleton regulating proteins (Blangy et al., 2020) in Tfr1-deficient osteoclast lineage cells relative to their control cells in our quantitative proteomic data bases (Supplementary files 1–3). More cytoskeleton proteins in mature osteoclasts were affected by Tfr1-dificiency than those in osteoclast precursors (Supplementary file 5). Among them, 25 cytoskeleton proteins were downregulated, whereas 17 proteins were upregulated by more than 1.2-log2fold in Tfr1-dificient osteoclasts compared to control cells. 15 osteoclast cytoskeleton-regulatory proteins remained the same between control and Tfr1-dificient osteoclasts. IPA analysis of the cytoskeleton-regulating pathways affected by Tfr1-deficiency in osteoclasts (Figure 9A) unveiled that the protein level of several molecules involved in activation of β_3_-integrin pathway, which is critical for osteoclast cytoskeleton organization and bone resorption (Teitelbaum, 2011), was decreased in Tfr1-null osteoclasts including β_3_-integrin (Itgb3), c-Src, Rap1a/b, Rap2b, Rapgef1, and Rap1gds (Supplementary file 5). A few actin-bundling and regulating proteins such as myosin heavy chain 14, α-actinin 1 and 4 (Actn1 and Actn4), and filamin A (Flna) were also downregulated in Tfr1-depleted osteoclasts. The branching and polymerization of filamentous actin in eukaryotic cells is mediated by the Arp2/3 complex which is controlled by the WRC. Mammalian WRC contains five subunits of Cyfip1/2, Hem1/2 (encoded by *Nckap1l* and *Nckap1*, respectively), Abi1/2/3, HSPC300, and WAVE1/2/3 (Rottner et al., 2021). The WRC is activated by the small GTPases Rac1 and Arf1 through their direct binding to Cyfip1/2 and Hem1/2. Strikingly, 12 out of 25 downregulated cytoskeletal proteins in Tfr1-null osteoclasts (Supplementary file 5) are the components of the Rac1/Arf-WRC-Arp2/3 axis. These data indicate that Tfr1-mediated iron uptake regulates osteoclast actin cytoskeleton organization, at least in part, through modulating the stability of the WRC-Arp2/3 actin-regulating module.

**Figure 9—figure supplement 1.**
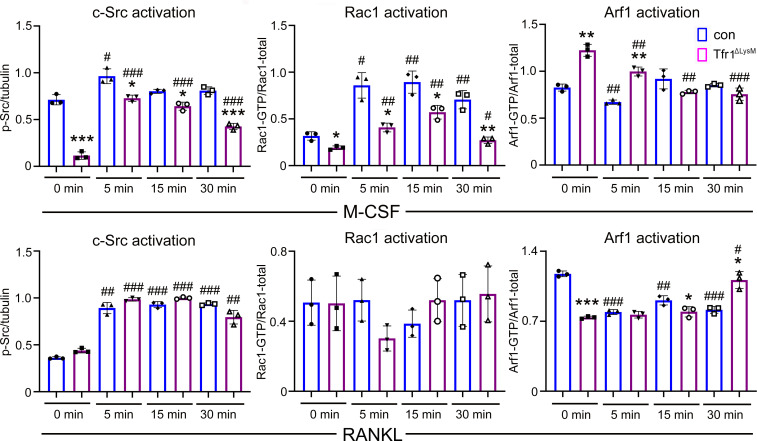
Quantification of immunoblotting bands in Figure 9B. Each band in immunoblots was quantified by densitometry using the NIH ImageJ software. n=3 from three immunoblots. * p<0.05, ** p<0.01, and p<0.001 vs control (con) of the same time point; The data are presented as mean ± SD. # p<0.05, ## p<0.01, and ### p<0.001 vs 0 min of the same genotype by one-way ANOVA. Figure 9—figure supplement 1—source data 1.Quantification of immunoblotting bands in Figure 9B.

To identify the mechanisms by which Tfr1 regulates osteoclast WRC complex and cytoskeleton, we examined the activation (phosphorylation) of protein tyrosine kinase c-Src which plays a pivotal role in osteoclast cytoskeleton organization downstream of integrin and M-CSF as well as RANKL signaling pathways in control and Tfr1^ΔlysM^ osteoclasts stimulated by M-CSF and RANKL. We also assayed the activation of small GTPases Rac1 and Arf1 that are upstream stimulators of the WRC complex in responses to M-CSF and RANKL in control and Tfr1^ΔlysM^ osteoclasts using GST-pulldown kits from Thermo-Fisher Scientific Inc. As shown in Figure 9B and Figure 9—figure supplement 1, while both M-CSF and RANKL activated c-Src in control and Tfr1^ΔlysM^ osteoclasts, the level of active (phosphorylated) c-Src in M-CSF, but not RANKL, stimulated Tfr1^ΔlysM^ osteoclasts was lower than control cells at all time points. Similarly, M-CSF induced Rac1 activation in form of Rac1-GTP was attenuated in Tfr1^ΔlysM^ osteoclasts compared to control cells at all time points, whereas RANKL did not arise Rac1-GTP level in control and Tfr1^ΔlysM^ osteoclasts. In regard to Arf1, M-CSF stimulation slightly decreased Arf1-GTP in both control and Tfr1^ΔlysM^ osteoclasts. The level of Arf1-GTP in Tfr1^ΔlysM^ osteoclasts was higher than control cells after starvation and 5 min of M-CSF stimulation. RANKL induced a mild decrease of Arf1-GTP in control osteoclasts and a little increase of Arf1-GTP in Tfr1^ΔlysM^ osteoclasts verse control cells after 30 min of RANKL incubation, although the Arf1-GTP was lower in starved and 15 min stimulated Tfr1^ΔlysM^ osteoclasts than control cells. These results indicate that loss of Tfr1 in osteoclasts influences M-CSF-mediated activation of the Src-Rac1-WRC axis, leading to disorganized cytoskeleton and compromised bone resorption.

To test whether overexpression of the components of WRC complex can rescue the cytoskeleton defect in Tfr1^ΔlysM^ osteoclasts, we constructed a recombinant retroviral vector expressing murine Hem1. After retroviral transduction in control and Tfr1-deleting BMM, we confirmed the expression of recombinant Hem1 by western blotting (Figure 9C). The overexpression of Hem1 enhanced the number of activated osteoclasts in control osteoclasts and partially rescued the cytoskeletal defect in Tfr1^ΔlysM^ osteoclasts (Figure 9D), suggesting that the other component(s) of the WRC complex or distinct pathways might also be involved in the Tfr1-mediated regulation of osteoclast cytoskeleton.

## Discussion

Loss of Tfr1 in osteoclast lineage cells results in >50% decrease in the total intracellular iron content in mature osteoclasts, whereas the iron levels in monocytes and pre-osteoclasts are not affected by Tfr1-deficiency (Figure 1). Since the heme transporter Hcp1 is upregulated during osteoclast differentiation and the fact that hemin but not FAC (absorbed through NTBI) is able to partially rescue the Tfr1-deficient osteoclast phenotypes (Figure 5), the residual cellular iron in Tfr1-deficient osteoclasts could be obtained through uptake of heme. Given that the key molecules involved in Tf-dependent and heme-dependent iron uptake pathway are significantly upregulated during osteoclast differentiation and that the expressions of NTBI iron transporter Zip14 and ferritin transporter (Scara-5 and Tim-2) are either decreased in osteoclasts or undetectable in osteoclast lineage cells (Figure 1A and data not shown), these results indicate that mature osteoclasts acquire extracellular iron largely through uptake of Tf and heme.

Although iron chelation by desferrioxamine has been shown to inhibit osteoclasts and prevent bone loss in ovariectomized mice (Ishii et al., 2009) and in a mouse model of Alzheimer’s disease (Guo et al.), the intrinsic functions of Tfr1 and Tfr1-mediated iron uptake in osteoclast lineage cells in vivo have not been investigated using genetically modified mouse models. To fill in this knowledge gap, we have generated two lines of *Tfrc* conditional knockout mice in which the Tfr1 expression is disrupted in *Lyz2*-Cre expressing myeloid osteoclast precursors and *Ctsk*-Cre expressing differentiated osteoclasts, respectively. The μCT analysis of control and Tfr1^ΔlysM^ mice at three different ages ranging from the pre-pubertal 3-week-old to the post-pubertal 10-week-old and the adult 6-month-old has unveiled a significant increase in trabecular bone mass in post-pubertal and adult female, but not male, Tfr1^ΔlysM^ mice compared to their age- and gender-matched control mice (Figure 2 and Figure 2—figure supplement 4). Although the significant increase of trabecular bone mass was observed in both genders of 10-week-old *Tfr1*^ΔCtsk^ mice, this phenotype is more pronounced in female knockout mice (Figure 2—figure supplement 6). These results indicate that the accrual of trabecular bone mass in Tfr1-deficient mice is gender-dependent. The exact mechanisms underlying the gender difference in the trabecular bone phenotype of mice upon the Tfr1-deficiency in osteoclasts are unknown and need further investigation since sex as a biological variable in mice and humans has been increasingly appreciated (Bhargava et al., 2021). A similar finding has been recently reported in *Slc2a1* (encoding the glucose transporter protein type 1, Glut1) myeloid conditional knockout mice (Li et al., 2020a). While Glut1-deficient BMM cultured from both male and female knockout mice have similar degree of blunted osteoclast differentiation in vitro, deletion of *Slc2a1* in *Lyz2*-Cre expressing myeloid cells in mice inhibits osteoclastogenesis and leads to increased trabecular bone mass in female but not male mice.

Estrogen has been reported to regulate systemic and cellular iron homeostasis by inhibiting the expression of hepcidin, a liver-derived iron regulating hormone that binds to and induces degradation of iron exporter Fpn (Hou et al., 2012; Yang et al., 2012; Ikeda et al., 2012); however, the levels of serum hepcidin and total iron are similar in control and Tfr1^ΔlysM^ male and female mice (Figure 2—figure supplement 3) excluding this possible mechanism. On the other hand, our in vitro supplemental experiment has demonstrated that 17-β estradiol inhibits the mRNA expression of *Tfrc* in mature osteoclasts and synergistically with Tfr1-deficiency attenuates the mRNA expression of *mt-Co1* (encoding the mitochondrial cytochrome c oxidase subunit I, mt-Co1), whereas dihydrotestosterone has no effects on Tfr1, Fpn, and mt-Co1 expression in osteoclast lineage cells (Figure 2—figure supplement 8). This finding is consistent with our recent published work that estrogen attenuates mitochondrial OXPHOS and ATP production in osteoclasts (Kim et al., 2020). The tissue- and bone cell type-specific sex differences in mitochondria and the underlying mechanisms in skeletons in both mice and humans need further investigation as recently reported in adipose tissue (Chella Krishnan et al., 2021).

It is well established that estrogen-mediated mechanisms play a key role in mediating bone accretion during pubertal growth period. During this developmental period, estrogen is known to exert an anabolic effect on osteoblast besides its well-established role in inhibiting osteoclast functions. However, the loss of estrogen as seen during menopause or due to ovariectomy in adults leads to bone loss primarily due to increased bone resorption. The increased trabecular bone volume of Tfr1^ΔLysM^ mice is evident at the post-pubertal age of 10 weeks but is not different at the pre-pubertal age of 3 weeks (Figure 2), thus suggesting that the skeletal phenotype gets manifested during pubertal and post-pubertal growth periods. While loss of Tfr1 does not affect estrogen deficiency-induced bone loss, the trabecular bone mass of Tfr1^ΔLysM^ mice is persistently higher than control mice after OVX. The issue of whether the skeletal phenotype in 10-week-old Tfr1^ΔLysM^ mice is dependent on pubertal increase in estrogen remains to be evaluated by performing OVX at pre-pubertal age of 3 weeks in future.

Both *Lyz2*-Cre and *Ctsk*-Cre mice are commonly used to conditionally delete the floxed genes at different stages of osteoclast lineage cells (Dallas et al., 2018). While the *Lyz2*-Cre deletes genes in monocytes of osteoclast precursor cells, it also targets other myeloid cells such as neutrophils and dendritic cells (Abram et al., 2014). Although Nfatc1-deletion by *Mx1*-Cre attenuates osteoclastogenesis and bone resorption in mice, Nfatc1^ΔlysM^ mice displayed no osteoclast and bone phenotypes (Aliprantis et al., 2008), suggesting that the deletion efficiency of *Lyz2*-Cre is weaker than *Mx1*-Cre and is allele-dependent (Dallas et al., 2018 and Clausen et al., 1999). The *Ctsk*-Cre, especially the *Ctsk*-Cre knock-in line, has been reported to delete genes at the late stages of differentiated osteoclasts (Nakamura et al., 2007). It should be noticed that the *Ctsk*-Cre has been reported to cause gene-disruption in mesenchymal progenitor cells including periosteal cells and osteoblasts (Yang et al., 2013 and Ruiz et al., 2016). Thus, the differences of skeletal phenotypes between Tfr1^ΔlysM^ and Tfr1^ΔCtsk^ mice might be caused by the in vivo efficacy and the specificity of targeting cells of these two Cre mouse lines.

The high bone mass observed in Tfr1^ΔlysM^ and Tfr1^ΔCtsk^ mice is more dramatic in the appendicular skeleton (femurs and tibias) (Figure 2 and Figure 2—figure supplement 6) than the axial skeleton (vertebras) (Figure 2—figure supplements 5 and 7). The trabecular bone is more vulnerable to Tfr1-deficiency than the cortical bone. The exact explanation(s) for distinct effects of Tfr1-deficiency on different sites and compartments of bone are not available. Our qPCR result demonstrated that deletion efficiency of Tfr1 gene, *Tfrc*, in vertebra is less than femurs (Figure 2—figure supplement 9). It is also likely that the different physical properties, remodeling rate, microenvironment, and the heterogeneous osteoclast subtypes in the distinct sites and compartments of bone may contribute to these site- and compartment-specific phenotypes which have been reported in other genetically modified mouse models (Cruz Grecco Teixeira et al., 2016 and Sato et al., 2020).

In this study, we have demonstrated that energy metabolism regulated by Tf11-mediated iron uptake is specifically indispensable for osteoclast activation and function but has little influence on osteoclast differentiation. In contrast, Ishii et al., 2009 have reported that in vitro knockdown of Tfr1 expression in BMM by short-hairpin RNA (shRNA) inhibits osteoclast differentiation. This discrepant finding might be caused by the off-target effects of Tfr1 shRNA on expression of genes essential for osteoclast differentiation or by the distinct effects of short-term Tfr1 downregulation by shRNA vs long-term genetic deletion of Tfr1 on osteoclast lineage cells. In support of our finding, it has been recently reported that deletion of mitochondria coactivator PGC-1β in myeloid osteoclast precursor cells by *Lyz2*-Cre diminishes mitochondrial biogenesis and function in osteoclasts, leading to cytoskeletal disorganization and bone resorption retardation with normal osteoclasts differentiation (Zhang et al., 2018). In this study, the PGC-1β-stimulated osteoclast cytoskeleton activation is identified to be mediated by GIT1 (G protein coupled receptor kinase 2 interacting protein 1) which has been reported to regulates osteoclast function and bone mass (Menon et al., 2010 and Jones and Katan, 2007). Another report by Nakano et al. has shown that deletion of G-protein Gα_13_ in myeloid cells augments mitochondrial biogenesis and metabolism in osteoclasts. The gain-of-function of mitochondrial pathway induced by Gα_13_-deletion in osteoclasts promotes actin cytoskeleton dynamic and bone resorption via activation of cytoskeleton regulators c-Src, Pyk2, and RhoA-Rock2. Again, Gα_13_-deletion in osteoclast lineage cells has minimum effects on osteoclastogenesis in vivo and in vitro (Nakano et al., 2019). Intriguingly, we have unveiled that Tfr1-mediated iron uptake regulates osteoclast cytoskeleton probably via a distinct mechanism. The level of GIT1, Rac1, Cdc42, and Rock2 remains unchanged in Tfr1-null osteoclasts (Supplementary file 5). However, the activation of c-Src-Rac1-WRC axis by M-CSF is attenuated and mostly downregulated in the absence of Tfr1, indicating that Tfr1 modulates osteoclast cytoskeleton through promoting the stability of WRC complex. In supporting of this premise, overexpression of Hem1 (also known as Nckap1l) partially rescues the cytoskeletal defects in Tfr1-deficient osteoclasts (Figure 9).

In summary, we have provided evidence demonstrating that Tfr1-mediated iron uptake is a major iron acquisition pathway in osteoclast lineage cells that differentially regulates trabecular bone remodeling in perpendicular and axial bones of female and male mice. The increased intracellular iron facilitated by Tfr1 is specifically required for osteoclast mitochondrial energy metabolism and cytoskeletal organization.

## Materials and methods

**Key resources table:** 

Reagent type (species) or resource	Designation	Source or reference	Identifiers	Additional information
Strain and strain background (*Mus musculus*)	*Tfrc*-flox	Dr. Nancy C Andrews		129Sv
Strain and strain background (*Mus musculus*)	*Tfrc*-flox	Zhao Lab		C57BL6/J
Strain and strain background (*Mus musculus*)	*Lyz2*-Cre	The Jackson Laboratory	cat#004781	C57BL6/J
Strain and strain background (*Mus musculus*)	*Ctsk*-Cre	Dr. Takashi Nakamura		C57BL6/J
Other	alpha-MEM	MilliporeSigma	M0644	cell culture medium
Other	Blasticidin	MilliporeSigma	203,350	Antibiotics (2 μg/ml)
Other	DMEM	MilliporeSigma	D-5648	cell culture medium
Other	Fetal bovine serum (FBS)	Hyclone		cell culture (10%)
Other	10× penicillin-streptomycin- l-glutamine	MilliporeSigma	G1146	Antibiotics (1×)
Other	10× trypsin/EDTA	Thermo-Fisher Scientific	15400–054	Antibiotics (1×)
Chemical compound and drug	Mouse apo-transferrin	MilliporeSigma	T0523	
Chemical compound and drug	Dihydrotestosterone	MilliporeSigma	D-073	10^–8^ M
Chemical compound and drug	Estrodiol	MilliporeSigma	E2257	10^–8^ M
Chemical compound and drug	ferric ammonium citrate (FAC)	MilliporeSigma	F5879	
Chemical compound and drug	Fe^59^ ferric chloride	PerkinElmer Inc		
Chemical compound and drug	Hemin	MilliporeSigma	H9039	
Chemical compound and drug	Sodium bicarbonate	MilliporeSigma	S5761	
Other	Albumin	MilliporeSigma	A7906	Blocking reagent (0.20% in PBS)
Other	Alexa Fluro-488 Phalloidin	Thermo-Fisher Scientific	A12379	Filament actin staining (1:400)
Other	Glycerol	MilliporeSigma	G5516	mounting reagent (80% in PBS)
Other	Hoechst 33,342	Thermo-Fisher Scientific	H3570	Nuclei staining (1:4,000 in PBS)
Other	Paraformaldehyde	MilliporeSigma	P6148	immunofluorescent staining (4% in PBS)
Other	Triton X-100	MilliporeSigma	T-9284	immunofluorescent staining (0.1%)
Other	NaK tartrate	MilliporeSigma	S6170	TRAP staining
Other	Naphthol AS-BI (phosphoric acid solution)	MilliporeSigma	1,802	TRAP staining
Other	3,3’-diaminobenzidine (DAB) tablets	MilliporeSigma	D-5905	pit staining
Other	30% H2O2	MilliporeSigma	216,763	pit staining
Other	Peroxidase-conjugated WGA (wheat germ agglutinin) lectin	MilliporeSigma	L-7017	pit staining (20 μg/ml)
Other	cOmplete EDTA-free protease inhibitor cocktail	MilliporeSigma	4693159001	protease inhibitor
Other	Enhanced chemiluminescent detection reagents (ECL)	MilliporeSigma	WBKLS0100	immunoblotting
Other	Polyvinylidene difluoride membrane (PVDF)	MilliporeSigma	IPVH00010	immunoblotting
Other	RIPA buffer	MilliporeSigma	R-0278	cell lysate
Antibody	Mouse monoclonal anti-cathepsin K (clone 182–12 G5)	MilliporeSigma	MAB3324	1:2000
Antibody	Mouse monoclonal anti-HA.11 (clone 16B12)	Biolegend	901,513	1:5000
Antibody	Mouse monoclonal anti-Tfr1 (clone H68.4)	Thermo-Fisher Scientific	13–6800	1:500
Antibody	Mouse monoclonal anti-tubulin (clone DM1A)	MilliporeSigma	T9026	1:3000
Antibody	Mouse monoclonal Total OXPHOS Antibody Cocktail	Abcam	ab110413	1:1000
Antibody	Goat polyclonal HRP-anti-mouse secondary antibody	Cell Signaling Technology	7,076	1:5000
Antibody	Goat polyclonal HRP-anti-rabbit secondary antibody	Cell Signaling Technology	7,074	1:5000
Commercial assay or kit	High-capacity cDNA reverse transcription kit	Thermo-Fisher Scientific	4368813	
Commercial assay or kit	RNeasy mini kit	Qiagen	74,104	
Commercial assay or kit	Serum TRAcP-5b	Immunodiagnostic Systems	SB-TR103	
Commercial assay or kit	RatLaps (CTx-I) EIA	Immunodiagnostic Systems	AC-06F1	
Commercial assay or kit	Rat/mouse PINP EIA	Immunodiagnostic Systems	AC-33F1	
Commercial assay or kit	Hepcidin Murine-Complete ELISA Kit	Intrinsic Lifesciences	HMC-001	
Commercial assay or kit	Pointe Scientific Iron/TIBC Reagents	Fisherscientific	23-666-320	
Commercial assay or kit	Iron Assay Kit	Abcam	ab83366	
Commercial assay or kit	Active Arf1 pull-down and detection kit	Thermo-Fisher Scientific	16,121	
Commercial assay or kit	Active Rac1 pull-down and detection kit	Thermo-Fisher Scientific	16,118	
Commercial assay or kit	TMTsixplex isobaric Mass Tagging kit	Thermo-Fisher Scientific	90,064	
Other	MitoTracker Green fluorescence	Thermo-Fisher Scientific	M7514	Mitochondrial assay reagent
Other	MitoSOX Red	Thermo-Fisher Scientific	M36008	Mitochondrial assay reagent
Other	JC-1 Dye	Thermo-Fisher Scientific	T3168	Mitochondrial assay reagent
Recombinant DNA reagent	Murine *Nckap1l*	Dharmacon Inc	MMM1013-202769283	cDNA template
Commercial assay or kit	TransIT-LT1	Mirus Bio LLC	MIR2300	DNA transfection reagent
Commercial assay or kit	*Acp5*	Thermo-Fisher Scientific	Mm00475698_m1	Quantitative PCR (qPCR) primer
Commercial assay or kit	*Ctsk*	Thermo-Fisher Scientific	Mm00484039_m1	qPCR primer
Commercial assay or kit	*mt-Cox1*	Thermo-Fisher Scientific	Mm00432648_m1	qPCR primer
Commercial assay or kit	*Dcstamp*	Thermo-Fisher Scientific	Mm04209236_m1	qPCR primer
Commercial assay or kit	*Nfatc1*	Thermo-Fisher Scientific	Mm00479445_m1	qPCR primer
Commercial assay or kit	*Mfsd7c*	Thermo-Fisher Scientific	Mm01302920_m1	qPCR primer
Commercial assay or kit	*Mrps2*	Thermo-Fisher Scientific	Mm00475529_m1	qPCR primer
Commercial assay or kit	*Runx2*	Thermo-Fisher Scientific	Mm00501584_m1	qPCR primer
Commercial assay or kit	*Slc11a2*	Thermo-Fisher Scientific	Mm00435363_m1	qPCR primer
Commercial assay or kit	*Slc39a14*	Thermo-Fisher Scientific	Mm01317439_m1	qPCR primer
Commercial assay or kit	*Slc40a1*	Thermo-Fisher Scientific	Mm01254822_m1	qPCR primer
Commercial assay or kit	*Slc46a1*	Thermo-Fisher Scientific	Mm00546630_m1	qPCR primer
Commercial assay or kit	*Tfrc*	Thermo-Fisher Scientific	Mm00441941_m1	qPCR primer
Commercial assay or kit	*Tfr2*	Thermo-Fisher Scientific	Mm00443703_m1	qPCR primer

The animal work follows the ARRIVE guidelines. All the in vivo and in vitro experiments were performed and analyzed in double-blinded manner.

### Mice and genotyping

*Tfrc*-flox congenic mice on a 129Sv background were kindly provided by Dr. Nancy C Andrews (Duke University). *Tfrc*-flox mice on a homogeneous C57BL/6 background were generated by backcrossing 129Sv *Tfr1*-flox mice with C57BL/6 mice for more than 10 generations. *Lyz2*-Cre mice on a C57BL/6 background (B6.129P2^tm1/cre^, stock number 004781) were purchased from The Jackson Laboratory (Bar Harbor, ME, USA). *Ctsk-*Cre congenic mice on a C57BL/6 background were obtained from Dr. Takashi Nakamura (Keio University, Tokyo, Japan). The primers and PCR protocols for genotyping *Tfrc*-flox and *Lyz2*-Cre mice followed those provided by The Jackson Laboratory. The following primers for genotyping *Ctsk-*Cre mice were used: P1N (5’-CCTAATTATTCCTTCCGCCAGGATG-3’), P2N (5’-CCAGGTTATGGGCAGAGATTTGCTT-3’), and P3N (5’-CACCGGCATCAACGTTTTCTTTTCG-3’). In vivo analyses of skeletal phenotypes were performed on F2 mice of mixed and homogeneous backgrounds.

### Reagents and antibodies

See Key resources table.

### Micro computed tomography

The left femurs, tibias, and L4 vertebrae were cleaned of soft tissues and fixed in 4% paraformaldehyde in PBS for overnight at 4°C. After washing with PBS for three times, the bones were stored in PBS with 0.02% sodium azide. The bones were loaded into a 12.3 mm diameter scanning tube and were imaged in a μCT (model μCT40, Scanco Medical). We integrated the scans into 3-D voxel images (1024×1024 pixel matrices for each individual planar stack) and used a Gaussian filter (sigma = 0.8 and support = 1) to reduce signal noise. A threshold of 200 was applied to all scans, at medium resolution (E = 55 kVp, I = 145 μA, and integration time = 200 ms).

### Histology and bone histomorphometry

The left femurs were embedded undecalcified in methyl methacrylate. The dynamic histomorphometric examination of trabecular bone formation was done on 5 μm longitudinal sections with a digitizer tablet (OsteoMetrics, Inc, Decatur, GA, USA) interfaced to a Zeiss Axioscope (Carl Zeiss, Thornwood, NY, USA) with a drawing tube attachment. The right femurs fixed and decalcified in 14% EDTA for 7–10 days. The bones were embedded in paraffin before obtaining 5 μm longitudinal sections. After removal of paraffin and rehydration, sections were stained for TRAP activity and counter-stained with fast green, and osteoclasts were enumerated on the trabecular bone surface using Osteomeasure histomorphometric software (OsteoMetrics, Inc, Decatur, GA, USA).

### Serum TRAcP-5b, CTx-I, and PINP ELISA

Blood was collected retro-orbitally under inhalation of a 2% isoflurane/oxygen mix anesthesia immediately prior to sacrifice. Serum was obtained by centrifugation of blood in a MiniCollect tube (catalog no. 450472, Greiner Bio-one GmbH, Austria). The serum levels of TRAcP-5b, CTx-I, and PINP were measured by a mouse TRAP (TRAcP 5b) kit (SB-TR103), RatLaps (CTx-I) EIA (AC-06F1), and rat/mouse PINP EIA kit (AC-33F1) from Immunodiagnostic Systems following their instructions.

### Serum hepcidin ELISA and serum total iron measurement

The serum level of hepcidin in control and Tfr1^ΔLysM^ mice was assayed by the Hepcidin Murine-Complete ELISA Kit from Intrinsic Lifesciences (La Jolla, CA, USA). The serum total iron concentration was measured using a Poine Scientific Iron kit.

### Ovariectomy

The 4.5-month-old control and Tfr1^ΔLysM^ female mice were anesthetized by a 0.5% isoflurane/oxygen mix and underwent bilateral OVX using the dorsal approach. The sham-operated mice had the ovaries exteriorized but not removed. Uterine weight was determined at sacrifice to verify the successful removal of the ovaries. 6 weeks after operation, mice were sacrificed, and femurs were isolated and fixed by 4% paraformaldehyde/PBS overnight before scanned by μCT.

### In vitro osteoclast cultures

Whole bone marrow was extracted from tibia and femurs of 6- to 8-week-old control and conditional knockout male and female mice. Red blood cells were lysed in buffer (150 mM NH4Cl, 10 mM KNCO3, 0.1 mM EDTA, and pH 7.4) for 5 min at room temperature. 5×10^6^ bone marrow cells were plated onto a 100 mm petri-dish and cultured in α–10 medium (α-MEM, 10% heat-inactivated fetal bovine serum [FBS], 1×PSG, penicillin-streptomycin-glutamine) containing 1/10 volume of CMG 14–12 (conditioned medium supernatant containing recombinant M-CSF at 1 μg/ml) for 4–5 days. BMM were lifted by 1×trypsin/EDTA and replated at density of 160/mm^2^ onto tissue culture plates or dishes with 1/100 vol of CMG 14–12 culture supernatant along for monocytes or cultured with 1/100 vol of CMG 14–12 culture supernatant plus 100 ng/ml of recombinant RANKL for 2 and 4 days to generate mononuclear pre-osteoclasts and mature osteoclasts, respectively.

### Retroviral transduction

A total of 3 μg of pMX (mammalian expression) retroviral vector or recombinant pMX vector expressing HA-tagged murine Hem1 was transfected into Plat E retroviral packing cells using TransIT-LT1 transfection reagent. Virus supernatants were collected at 48 hr after transfection. 5×10^6^ bone marrow cells isolated from Tfr1 myeloid conditional knockout mice were plated onto a 100 mm petri-dish and cultured in α–10 medium (α-MEM, 10% heat-inactivated FBS, 1×PSG) containing 1/10 volume of CMG 14–12 supernatant for two days. BMM were transduced with viruses for 24 hr in α–10 medium containing 1/10 volume of CMG 14–12 supernatant and 20 μg/ml of protamine. Cells were then lifted by 1×trypsin/EDTA and replated at 2×10^6^ density onto a 100 mm petri-dish. The positively transduced cells were selected in α–10 medium containing M-CSF and 1.5 μg/ml of blasticidin for 3 days.

### TRAP staining

Osteoclasts cultured on 48-well tissue culture plate were fixed with 4% paraformaldehyde/PBS for 20 min at room temperature. After washing with PBS for 5 min twice, TRAP was stained with NaK tartrate and naphthol AS-BI phosphoric acid. Photomicrographs were taken with a stereomicroscope with a digital camera (Discovery V12 and AxioCam; Carl Zeiss, Inc). The number of osteoclasts with more than three nuclei was counted and analyzed by GraphPad Prism 6 in a double-blinded manner.

### Resorption pit staining

Mature osteoclasts grown on cortical bovine bone slices were fixed with 4% paraformaldehyde/PBS for 20 min. After washing in PBS for 5 min twice, cells were removed from bone slices with a soft brush. The slices were then incubated with 20 µg/ml peroxidase-conjugated wheat germ agglutinin lectin for 60 min at room temperature. After washing in PBS twice, bone chips were incubated with 0.52 mg/ml 3,3_-diaminobenzidine and 0.03% H2O2 for 30 min. Samples were mounted with 80% glycerol/PBS and photographed with Zeiss AxioPlan2 microscope equipped with an Olympus DP73 digital camera. The resorbed area/bone slice was quantified by ImageJ software (National Institutes of Health), and the percentage of pit area vs that of the whole bone slice was calculated and analyzed by GraphPad Prism 6.

### Fluorescent staining of actin filaments and nuclei

Osteoclasts cultured on glass coverslips or bone slices were fixed with 4% paraformaldehyde in PBS for 20 min and permeabilized with 0.1% Triton X-100/PBS for 10 min at room temperature. The filament actin and nuclei were labeled by Alexa-488 conjugated Phalloidin (1:100 from a 1 mg/ml stock) and Hoechst 33,342 (1:4000 from a 10 mg/ml stock), respectively, for 15 min at room temperature. After two times 5-min wash with PBS, samples were mounted with 80% glycerol/PBS and photographed under a Zeiss AxioImager Z1 fluorescent microscope equipped with Zeiss AxioCam MRm monochromatic and MR5c color cameras with a set of fluorescent filters. The percentage of active osteoclasts (podosome-belt bearing osteoclasts on glass coverslips and actin-ring bearing osteoclasts on bone slices) out of total osteoclasts was calculated and analyzed by GraphPad Prism 6.

### RNA isolation and real-time qPCR

Total RNA was purified using RNeasy mini kit (Qiagen) according to the manufacture’s protocol. First-strand cDNAs were synthesized from 0.5 to 1 μg of total RNA using the High-Capacity cDNA Reverse Transcription kits (Thermo-Fisher Scientific) following the manufacturer’s instructions. TaqMan real-time qPCR was performed using the primers from Thermo-Fisher Scientific (Key resources table). Samples were amplified using the StepOnePlus real-time PCR system (Life Technologies) with an initial denaturation at 95°C for 10 min, followed by 40 cycles of 95°C for 15 s and 60°C for 1 min. The relative cDNA amount was calculated by normalizing to that of the mitochondrial gene Mrps2, which is steadily expressed in both BMM and osteoclasts, using the ΔCt method.

### Immunoblotting

Cells were washed with ice-cold PBS twice and lysed in 1× RIPA buffer containing cOmplete Mini EDTA-free protease inhibitor cocktail. After incubation on ice for 30 min, cell lysates were clarified by centrifugation at 14,000 rpm for 15 min at 4°C. 10–30 μg of total protein was subjected to 8 or 10% SDS-PAGE gels and transferred electrophoretically onto polyvinylidene difluoride membrane by a semi-dry blotting system (Bio-Rad). The membrane was blocked in 5% fat-free milk/Tris-buffered saline for 1 hr and incubated with primary antibodies at 4°C overnight followed by horseradish peroxidase conjugated secondary antibodies. After rinsing three times with Tris-buffered saline containing 0.1% Tween 20, the membrane was incubated with enhanced chemiluminescent for 5 min.

### ^59^Fe-transferrin uptake and colorimetric total cellular iron measurement

Mouse apo-transferrin was labeled with ^59^Fe and gel-filtered on a Sephadex G-50 column. For Tf-dependent ^59^Fe influx measurements, 25 µg of ^59^Fe-labeled Tf was added to each well of a 6-well plate with 3 ml of medium and incubated in a CO2 incubator (37°C, 5% CO2) with rotation at 80 rpm. At various times after adding labeled Tf, the medium was aspirated, and cells were washed gently three times with 2 ml of cold PBS. Wells were extracted with 1 ml of 0.1 N NaOH; radioactivity was determined on a 0.5 ml aliquot by a gamma counter. Protein was determined on 25 µl of the extract.

To measure cellular iron, cells cultured in 6-well plates were collected in 50 µl of the Iron Assay Buffer provided in an Iron Assay kit (Abcam) and were homogenized with pellet pestles associated with a motor. The cells lysates were centrifuged at 15,000 rpm for 10 min. The supernatants proceeded to measure the total iron (Fe^2+^ and Fe^3+^) concentration following the manufacturer’s protocol.

### Quantitative proteomic and mass-spectrometry

Cells were harvested and lysed in 2% SDS lysis buffer. A total of 100 µg of proteins in each cell lysate was reduced, alkylated, and digested using filter-aided sample preparation. Tryptic peptides were cleaned by solid phase extraction (SPE), normalized and labeled using a TMTsixplex isobaric Mass Tagging kit. The labeled peptides were cleaned by SPE and mixed. The mixed peptides from each cell culture were separated into 36 fractions on a 100 × 1.0 mm Acquity BEH C18 column (Waters) using an UltiMate 3000 UHPLC system (Thermo-Fisher Scientific) with a 40 min gradient from 99:1 to 60:40 buffer A:B (Buffer A contains 0.5% acetonitrile and 10 mM ammonium hydroxide). Buffer B contains 10 mM ammonium hydroxide in acetonitrile ratio under basic (pH 10) conditions and then consolidated into 13 super-fractions.

Each super-fraction was then further separated by reverse phase XSelect CSH C18 2.5 mm resin (Waters) on an in-line 150 × 0.075 mm column using an UltiMate 3000 RSLCnano system (Thermo). Peptides were eluted using a 60 min gradient from 97:3 to 60:40 buffer A:B ratio. Here, buffer A contains 0.1% formic acid and 0.5% acetonitrile, and buffer B contains 0.1% formic acid and 99.9% acetonitrile. Eluted peptides were ionized by electrospray (2.15 kV) followed by mass spectrometric analysis on an Orbitrap Fusion Lumos mass spectrometer (Thermo) using multi-notch MS3 parameters. The mass spectrometry data were acquired using the FTMS (fourier transform mass spectrometer) analyzer in top-speed profile mode at a resolution of 120,000 over a range of 375–1500 m/z. Following CID activation with normalized collision energy of 35.0, MS/MS data were acquired using the ion trap analyzer in centroid mode and normal mass range. Using synchronous precursor selection, up to 10 MS/MS precursors were selected for HCD activation with normalized collision energy of 65.0, followed by acquisition of MS3 reporter ion data using the FTMS analyzer in profile mode at a resolution of 50,000 over a range of 100–500 m/z.

Proteins were identified and reporter ions quantified by searching the UniprotKB Mouse database using MaxQuant (version 1.6.10.43, Max Planck Institute) with a parent ion tolerance of 3 ppm, a fragment ion tolerance of 0.5 Da, a reporter ion tolerance of 0.001 Da, trypsin/P enzyme with two missed cleavages, variable modifications including oxidation on M and acetyl on protein N-term, and fixed modification of carbamidomethyl on C. Protein identifications were accepted if they could be established with less than 1.0% false discovery. Proteins identified only by modified peptides were removed. Protein probabilities were assigned by the Protein Prophet algorithm. TMT MS3 reporter ion intensity values were analyzed for changes in total protein.

### Mitochondrial mass, ROS production, and membrane potential measurements

Mitochondrial content, mitochondria-derived ROS, and mitochondrial membrane potential were measured using MitoTracker Green fluorescence, MitoSOX Red Mitochondrial Superoxide Indicator, and JC-1 Dye from Thermo-Fisher Scientific, respectively, using fluorescence microscopy. Briefly, cells cultured onto glass-bottom imaging dishes were incubated with MitoTracker Green (50 nM), MitoSOX (5 µM), or JC-1 (5 µg/ml), respectively, for 15 min at 37°C. The probes were then washed off, and cells were examined under fluorescent microscope. The epifluorescent images of five randomly chosen areas per group (control or knockout) were taken in a blinded fashion. Each area had between 5 and 10 cells. The images were analyzed with Image J software also in a blinded fashion. The fluorescent intensities of cells were individually measured, and the results were presented as mean fluorescent intensity per cell in arbitrary units.

### Seahorse mitochondrial flux analysis

BMM were plated in wells of Seahorse XF96 cell culture plates. The cells were cultured with 10 ng/ml M-CSF alone or 10 ng/ml M-CSF plus 100 ng/ml recombinant RANKL for 2 days to generate monocytes and pre-osteoclasts or 4 days for mature osteoclasts. On the day of respiratory function measurements, the media in the wells were changed to unbuffered Dulbecco’s modified Eagle’s medium supplemented with 4 mM glutamate and incubated in a non-CO2 incubator for 1 hr at 37°C. Three baseline measurements were acquired before injection of mitochondrial inhibitors or uncouplers. OCR measurements were taken after sequential addition of oligomycin (10 µM), FCCP (5 µM), and rotenone/antimycin A mixture (10 µM). OCRs were calculated by the Seahorse XF-96 software and represent an average of three measurements on 18–24 different wells. The rate of measured oxygen consumption was reported as fmol of O2 consumed per min per cell.

### Rac1 and Arf1 activation assay

BMM obtained from the control and Tfr1^ΔLysM^ mice were cultured with M-CSF and RANKL for 3 days to generate pre-osteoclasts. The cells were then under serum and cytokine starvation for 6 hr before stimulated with 50 ng/ml M-CSF and 100 ng/ml RANKL, respectively. The activation of small GTPases Rac1 and Arf1 was assayed by GST-pull down assay kits from Thermo-Fisher Scientific Inc.

### Statistics

Based on power analysis using SD and the population variance estimated from our previous studies and reports by others, the required sample size of mice for *α*=0.05 (two-sided) and a power of 0.95 is ≥6 animals per group. The in vivo biological replicates are defined as an individual mouse for each experiment. All in vitro data were representatives of individual biological replicates from independent experiments and not technical replicates (repeated measurements of the same sample). For all graphs, data are represented as the mean ± SD. For comparison of two groups, data were analyzed using a two-tailed Student’s t-test. For comparison of more than two groups, data were analyzed using one-way ANOVA, and the Bonferroni procedure was used for Tukey comparison. For all statistical tests, the analysis was performed using Prism 6 (GraphPad Software, La Jolla, CA), and a p value of less than 0.05 was considered significant.

### Study approval

All animal protocols and procedures used in animal studies were approved by the Institutional Animal Care and Use Committees of the University of Arkansas for Medical Sciences, Long Beach VA Healthcare System, and Loma Linda VA Healthcare System (IACUC #1,685 and #1774). The protocols for generation and use of recombinant DNAs and retroviruses were approved by Institutional Biosafety Committee of Long Beach VA Healthcare System (approval #1774).

## Data Availability

All data generated or analysed during this study are included in the manuscript and supporting file.

## Additional files

Supplementary file 1. Bone marrow monocytes qProteomics.

Supplementary file 2. Pre-osteoclasts qProteomics.

Supplementary file 3. Osteoclasts qProteomics.

Supplementary file 4. Ingenuity pathway analysis (IPA) of canonical pathways in osteoclasts.

Supplementary file 5. Changes of cytoskeletal proteins in TfR1-null osteoclast lineage cells revealed by qProteomics.

MDAR checklist

## References

[bib1] Abram CL, Roberge GL, Hu Y, Lowell CA (2014). Comparative analysis of the efficiency and specificity of myeloid-Cre deleting strains using ROSA-EYFP reporter mice. Journal of Immunological Methods.

[bib2] Aliprantis AO, Ueki Y, Sulyanto R, Park A, Sigrist KS, Sharma SM, Ostrowski MC, Olsen BR, Glimcher LH (2008). NFATc1 in mice represses osteoprotegerin during osteoclastogenesis and dissociates systemic osteopenia from inflammation in cherubism. The Journal of Clinical Investigation.

[bib3] Almeida A, Roberts I (2005). Bone involvement in sickle cell disease. British Journal of Haematology.

[bib4] Arnett TR, Orriss IR (2018). Metabolic properties of the osteoclast. Bone.

[bib5] Balogh E, Paragh G, Jeney V (2018). Influence of iron on bone homeostasis. Pharmaceuticals.

[bib6] Barrientos T, Laothamatas I, Koves TR, Soderblom EJ, Bryan M, Moseley MA, Muoio DM, Andrews NC (2015). Metabolic catastrophe in mice lacking transferrin receptor in muscle. EBioMedicine.

[bib7] Bhargava A, Arnold AP, Bangasser DA, Denton KM, Gupta A, Hilliard Krause LM, Mayer EA, McCarthy M, Miller WL, Raznahan A, Verma R (2021). Considering sex as a biological variable in basic and clinical studies. An Endocrine Society Scientific Statement Endocr Rev.

[bib8] Blangy A, Bompard G, Guerit D, Marie P, Maurin J, Morel A, Vives V (2020). The osteoclast cytoskeleton - current understanding and therapeutic perspectives for osteoporosis. Journal of Cell Science.

[bib9] Boyle WJ, Simonet WS, Lacey DL (2003). Osteoclast differentiation and activation. Nature.

[bib10] Camaschella C, Roetto A, Calì A, De Gobbi M, Garozzo G, Carella M, Majorano N, Totaro A, Gasparini P (2000). The gene TFR2 is mutated in a new type of haemochromatosis mapping to 7q22. Nature Genetics.

[bib11] Chella Krishnan K, Vergnes L, Acín-Pérez R, Stiles L, Shum M, Ma L, Mouisel E, Pan C, Moore TM, Péterfy M, Romanoski CE, Reue K, Björkegren JLM, Laakso M, Liesa M, Lusis AJ (2021). Sex-specific genetic regulation of adipose mitochondria and metabolic syndrome by Ndufv2. Nature Metabolism.

[bib12] Chen AC, Donovan A, Ned-Sykes R, Andrews NC (2015). Noncanonical role of transferrin receptor 1 is essential for intestinal homeostasis. PNAS.

[bib13] Ch’uan CH (1931). Mitochondria in osteoclasts. The Anatomical Record.

[bib14] Clausen BE, Burkhardt C, Reith W, Renkawitz R, Förster I (1999). Conditional gene targeting in macrophages and granulocytes using LysMcre mice. Transgenic Research.

[bib15] Cruz Grecco Teixeira MB, Martins GM, Miranda-Rodrigues M, De Araújo IF, Oliveira R, Brum PC, Azevedo Gouveia CH (2016). lack of α2C-adrenoceptor results in contrasting phenotypes of long bones and vertebra and prevents the thyrotoxicosis-induced osteopenia. PLOS ONE.

[bib16] Dallas SL, Xie Y, Shiflett LA, Ueki Y (2018). Mouse Cre models for the study of bone diseases. Current Osteoporosis Reports.

[bib17] Dede AD, Trovas G, Chronopoulos E, Triantafyllopoulos IK, Dontas I, Papaioannou N, Tournis S (2016). Thalassemia-associated osteoporosis: a systematic review on treatment and brief overview of the disease. Osteoporosis International.

[bib18] Diaz-Castro J, López-Frías MR, Campos MS, López-Frías M, Alférez MJM, Nestares T, Ojeda ML, López-Aliaga I (2012). Severe nutritional iron-deficiency anaemia has a negative effect on some bone turnover biomarkers in rats. European Journal of Nutrition.

[bib19] Donovan A, Roy CN, Andrews NC (2006). The ins and outs of iron homeostasis. Physiology.

[bib20] Fleming RE, Ahmann JR, Migas MC, Waheed A, Koeffler HP, Kawabata H, Britton RS, Bacon BR, Sly WS (2002). Targeted mutagenesis of the murine transferrin receptor-2 gene produces hemochromatosis. PNAS.

[bib21] Fujiwara T, Ye S, Castro-Gomes T, Winchell CG, Andrews NW, Voth DE, Varughese KI, Mackintosh SG, Feng Y, Pavlos N, Nakamura T, Manolagas SC, Zhao H (2016). PLEKHM1/DEF8/RAB7 complex regulates lysosome positioning and bone homeostasis. JCI Insight.

[bib22] Fung EB, Harmatz PR, Milet M, Coates TD, Thompson AA, Ranalli M, Mignaca R, Scher C, Giardina P, Robertson S, Neumayr L, Vichinsky EP, Multi-Center Iron Overload Study Group (2008). Fracture prevalence and relationship to endocrinopathy in iron overloaded patients with sickle cell disease and thalassemia. Bone.

[bib23] Gammella E, Buratti P, Cairo G, Recalcati S (2017). The transferrin receptor: the cellular iron gate. Metallomics : Integrated Biometal Science.

[bib24] Gu Z, Wang H, Xia J, Yang Y, Jin Z, Xu H, Shi J, De Domenico I, Tricot G, Zhan F (2015). Decreased ferroportin promotes myeloma cell growth and osteoclast differentiation. Cancer Research.

[bib25] Guggenbuhl P, Fergelot P, Doyard M, Libouban H, Roth MP, Gallois Y, Chalès G, Loréal O, Chappard D (2011). Bone status in a mouse model of genetic hemochromatosis. Osteoporosis International.

[bib26] Guo JP, Pan JX, Xiong L, Xia WF, Cui S, Xiong WC (2015). Iron chelation inhibits osteoclastic differentiation in vitro and in Tg2576 mouse model of alzheimer’s disease. PLOS ONE.

[bib27] Hentze MW, Muckenthaler MU, Galy B, Camaschella C (2010). Two to tango: regulation of Mammalian iron metabolism. Cell.

[bib28] Holtrop ME, King GJ (1977). The ultrastructure of the osteoclast and its functional implications. Clinical Orthopaedics and Related Research.

[bib29] Hou Y, Zhang S, Wang L, Li J, Qu G, He J, Rong H, Ji H, Liu S (2012). Estrogen regulates iron homeostasis through governing hepatic hepcidin expression via an estrogen response element. Gene.

[bib30] Ikeda Y, Tajima S, Izawa-Ishizawa Y, Kihira Y, Ishizawa K, Tomita S, Tsuchiya K, Tamaki T (2012). Estrogen regulates hepcidin expression via GPR30-BMP6-dependent signaling in hepatocytes. PLOS ONE.

[bib31] Indo Y, Takeshita S, Ishii KA, Hoshii T, Aburatani H, Hirao A, Ikeda K (2013). Metabolic regulation of osteoclast differentiation and function. Journal of Bone and Mineral Research.

[bib32] Ishii K, Fumoto T, Iwai K, Takeshita S, Ito M, Shimohata N, Aburatani H, Taketani S, Lelliott CJ, Vidal-Puig A, Ikeda K (2009). Coordination of PGC-1beta and iron uptake in mitochondrial biogenesis and osteoclast activation. Nature Medicine.

[bib33] Jabara HH, Boyden SE, Chou J, Ramesh N, Massaad MJ, Benson H, Bainter W, Fraulino D, Rahimov F, Sieff C, Liu ZJ, Alshemmari SH, Al-Ramadi BK, Al-Dhekri H, Arnaout R, Abu-Shukair M, Vatsayan A, Silver E, Ahuja S, Davies EG, Sola-Visner M, Ohsumi TK, Andrews NC, Notarangelo LD, Fleming MD, Al-Herz W, Kunkel LM, Geha RS (2016). A missense mutation in TFRC, encoding transferrin receptor 1, causes combined immunodeficiency. Nature Genetics.

[bib34] Jandl NM, Rolvien T, Schmidt T, Mussawy H, Nielsen P, Oheim R, Amling M, Barvencik F (2020). Impaired bone microarchitecture in patients with hereditary hemochromatosis and skeletal complications. Calcified Tissue International.

[bib35] Jin Z, Wei W, Yang M, Du Y, Wan Y (2014). Mitochondrial complex I activity suppresses inflammation and enhances bone resorption by shifting macrophage-osteoclast polarization. Cell Metabolism.

[bib36] Jones NP, Katan M (2007). Role of phospholipase Cgamma1 in cell spreading requires association with a beta-Pix/GIT1-containing complex, leading to activation of Cdc42 and Rac1. Molecular and Cellular Biology.

[bib37] Katsumata S, Katsumata-Tsuboi R, Uehara M, Suzuki K (2009). Severe iron deficiency decreases both bone formation and bone resorption in rats. The Journal of Nutrition.

[bib38] Kawabata H (2019). Transferrin and transferrin receptors update. Free Radical Biology & Medicine.

[bib39] Kim JM, Jeong D, Kang HK, Jung SY, Kang SS, Min BM (2007). Osteoclast precursors display dynamic metabolic shifts toward accelerated glucose metabolism at an early stage of RANKL-stimulated osteoclast differentiation. Cellular Physiology and Biochemistry.

[bib40] Kim H, Kim T, Jeong BC, Cho IT, Han D, Takegahara N, Negishi-Koga T, Takayanagi H, Lee JH, Sul JY, Prasad V, Lee SH, Choi Y (2013). Tmem64 modulates calcium signaling during RANKL-mediated osteoclast differentiation. Cell Metabolism.

[bib41] Kim H, Lee YD, Kim HJ, Lee ZH, Kim HH (2017). SOD2 and Sirt3 control osteoclastogenesis by regulating mitochondrial ROS. Journal of Bone and Mineral Research.

[bib42] Kim H-N, Ponte F, Nookaew I, Ucer Ozgurel S, Marques-Carvalho A, Iyer S, Warren A, Aykin-Burns N, Krager K, Sardao VA, Han L, de Cabo R, Zhao H, Jilka RL, Manolagas SC, Almeida M (2020). Estrogens decrease osteoclast number by attenuating mitochondria oxidative phosphorylation and ATP production in early osteoclast precursors. Scientific Reports.

[bib43] Kubatzky KF, Uhle F, Eigenbrod T (2018). From macrophage to osteoclast - How metabolism determines function and activity. Cytokine.

[bib44] Levy JE, Jin O, Fujiwara Y, Kuo F, Andrews NC (1999). Transferrin receptor is necessary for development of erythrocytes and the nervous system. Nature Genetics.

[bib45] Li B, Lee WC, Song C, Ye L, Abel ED, Long F (2020a). Both aerobic glycolysis and mitochondrial respiration are required for osteoclast differentiation. The FASEB Journal.

[bib46] Li J, Pan X, Pan G, Song Z, He Y, Zhang S, Ye X, Yang X, Xie E, Wang X, Mai X, Yin X, Tang B, Shu X, Chen P, Dai X, Tian Y, Yao L, Han M, Xu G, Zhang H, Sun J, Chen H, Wang F, Min J, Xie L (2020b). Transferrin receptor 1 regulates thermogenic capacity and cell fate in brown/beige adipocytes. Advanced Science.

[bib47] Matak P, Matak A, Moustafa S, Aryal DK, Benner EJ, Wetsel W, Andrews NC (2016). Disrupted iron homeostasis causes dopaminergic neurodegeneration in mice. PNAS.

[bib48] Mbalaviele G, Novack DV, Schett G, Teitelbaum SL (2017). Inflammatory osteolysis: a conspiracy against bone. The Journal of Clinical Investigation.

[bib49] Menon P, Yin G, Smolock EM, Zuscik MJ, Yan C, Berk BC (2010). GPCR kinase 2 interacting protein 1 (GIT1) regulates osteoclast function and bone mass. Journal of Cellular Physiology.

[bib50] Nakamura T, Imai Y, Matsumoto T, Sato S, Takeuchi K, Igarashi K, Harada Y, Azuma Y, Krust A, Yamamoto Y, Nishina H, Takeda S, Takayanagi H, Metzger D, Kanno J, Takaoka K, Martin TJ, Chambon P, Kato S (2007). Estrogen prevents bone loss via estrogen receptor alpha and induction of Fas ligand in osteoclasts. Cell.

[bib51] Nakano S, Inoue K, Xu C, Deng Z, Syrovatkina V, Vitone G, Zhao L, Huang XY, Zhao B (2019). G-protein Gα13 functions as a cytoskeletal and mitochondrial regulator to restrain osteoclast function. Scientific Reports.

[bib52] Nakashima T, Hayashi M, Takayanagi H (2012). New insights into osteoclastogenic signaling mechanisms. Trends in Endocrinology and Metabolism.

[bib53] Novack DV, Teitelbaum SL (2008). The osteoclast: Friend or foe?. Annual Review of Pathology.

[bib54] Pantopoulos K, Porwal SK, Tartakoff A, Devireddy L (2012). Mechanisms of mammalian iron homeostasis. Biochemistry.

[bib55] Rauner M, Baschant U, Roetto A, Pellegrino RM, Rother S, Salbach-Hirsch J, Weidner H, Hintze V, Campbell G, Petzold A, Lemaitre R, Henry I, Bellido T, Theurl I, Altamura S, Colucci S, Muckenthaler MU, Schett G, Komla-Ebri DSK, Bassett JHD, Williams GR, Platzbecker U, Hofbauer LC (2019). Transferrin receptor 2 controls bone mass and pathological bone formation via BMP and Wnt signalling. Nature Metabolism.

[bib56] Rishi G, Secondes ES, Wallace DF, Subramaniam VN (2016). Normal systemic iron homeostasis in mice with macrophage-specific deletion of transferrin receptor 2. American Journal of Physiology. Gastrointestinal and Liver Physiology.

[bib57] Rottner K, Stradal TEB, Chen B (2021). WAVE regulatory complex. Current Biology.

[bib58] Ruiz P, Martin-Millan M, Gonzalez-Martin MC, Almeida M, González-Macias J, Ros MA (2016). CathepsinKCre mediated deletion of βcatenin results in dramatic loss of bone mass by targeting both osteoclasts and osteoblastic cells. Scientific Reports.

[bib59] Sato T, Iwata T, Usui M, Kokabu S, Sugamori Y, Takaku Y, Kobayashi T, Ito K, Matsumoto M, Takeda S, Xu R, Chida D (2020). Bone phenotype in melanocortin 2 receptor-deficient mice. Bone Reports.

[bib60] Senyilmaz D, Virtue S, Xu X, Tan CY, Griffin JL, Miller AK, Vidal-Puig A, Teleman AA (2015). Regulation of mitochondrial morphology and function by stearoylation of TFR1. Nature.

[bib61] Siddiqui JA, Partridge NC (2016). Physiological bone remodeling: Systemic regulation and growth factor involvement. Physiology.

[bib62] Srinivasan S, Koenigstein A, Joseph J, Sun L, Kalyanaraman B, Zaidi M, Avadhani NG (2010). Role of mitochondrial reactive oxygen species in osteoclast differentiation. Annals of the New York Academy of Sciences.

[bib63] Teitelbaum SL (2011). The osteoclast and its unique cytoskeleton. Annals of the New York Academy of Sciences.

[bib64] Tsay J, Yang Z, Ross FP, Cunningham-Rundles S, Lin H, Coleman R, Mayer-Kuckuk P, Doty SB, Grady RW, Giardina PJ, Boskey AL, Vogiatzi MG (2010). Bone loss caused by iron overload in a murine model: importance of oxidative stress. Blood.

[bib65] Väänänen HK, Zhao H, Mulari M, Halleen JM (2000). The cell biology of osteoclast function. Journal of Cell Science.

[bib66] Walker DG (1975). Control of bone resorption by hematopoietic tissue. The induction and reversal of congenital osteopetrosis in mice through use of bone marrow and splenic transplants. The Journal of Experimental Medicine.

[bib67] Wang L, Fang B, Fujiwara T, Krager K, Gorantla A, Li C, Feng JQ, Jennings ML, Zhou J, Aykin-Burns N, Zhao H (2018). Deletion of ferroportin in murine myeloid cells increases iron accumulation and stimulates osteoclastogenesis *in vitro* and *in vivo*. The Journal of Biological Chemistry.

[bib68] Wang S, He X, Wu Q, Jiang L, Chen L, Yu Y, Zhang P, Huang X, Wang J, Ju Z, Min J, Wang F (2020). Transferrin receptor 1-mediated iron uptake plays an essential role in hematopoiesis. Haematologica.

[bib69] Xiao W, Beibei F, Guangsi S, Yu J, Wen Z, Xi H, Youjia X (2015). Iron overload increases osteoclastogenesis and aggravates the effects of ovariectomy on bone mass. The Journal of Endocrinology.

[bib70] Xiao Y, Palomero J, Grabowska J, Wang L, de Rink I, van Helvert L, Borst J (2017). Macrophages and osteoclasts stem from a bipotent progenitor downstream of a macrophage/osteoclast/dendritic cell progenitor. Blood Advances.

[bib71] Xu W, Barrientos T, Andrews NC (2013). Iron and copper in mitochondrial diseases. Cell Metabolism.

[bib72] Xu W, Barrientos T, Mao L, Rockman HA, Sauve AA, Andrews NC (2015). Lethal cardiomyopathy in mice lacking transferrin receptor in the heart. Cell Reports.

[bib73] Yang Q, Jian J, Katz S, Abramson SB, Huang X (2012). 17β-Estradiol inhibits iron hormone hepcidin through an estrogen responsive element half-site. Endocrinology.

[bib74] Yang W, Wang J, Moore DC, Liang H, Dooner M, Wu Q, Terek R, Chen Q, Ehrlich MG, Quesenberry PJ, Neel BG (2013). Ptpn11 deletion in a novel progenitor causes metachondromatosis by inducing hedgehog signalling. Nature.

[bib75] Zaidi M (2007). Skeletal remodeling in health and disease. Nature Medicine.

[bib76] Zhang Y, Rohatgi N, Veis DJ, Schilling J, Teitelbaum SL, Zou W (2018). PGC1β organizes the osteoclast cytoskeleton by mitochondrial biogenesis and activation. Journal of Bone and Mineral Research.

[bib77] Zhao H (2012). Membrane trafficking in osteoblasts and osteoclasts: new avenues for understanding and treating skeletal diseases. Traffic.

[bib78] Zhou J, Ye S, Fujiwara T, Manolagas SC, Zhao H (2013). Steap4 plays a critical role in osteoclastogenesis in vitro by regulating cellular iron/reactive oxygen species (ROS) levels and cAMP response element-binding protein (CREB) activation. The Journal of Biological Chemistry.

