# Supplementary figures and images for: Transferrin receptor 1-mediated iron uptake regulates bone mass in mice via osteoclast mitochondria and cytoskeleton
Source: eLife. 2022 Jun 27;11:e73539. doi: 10.7554/eLife.73539 (PMC9352353; doi:10.7554/eLife.73539)

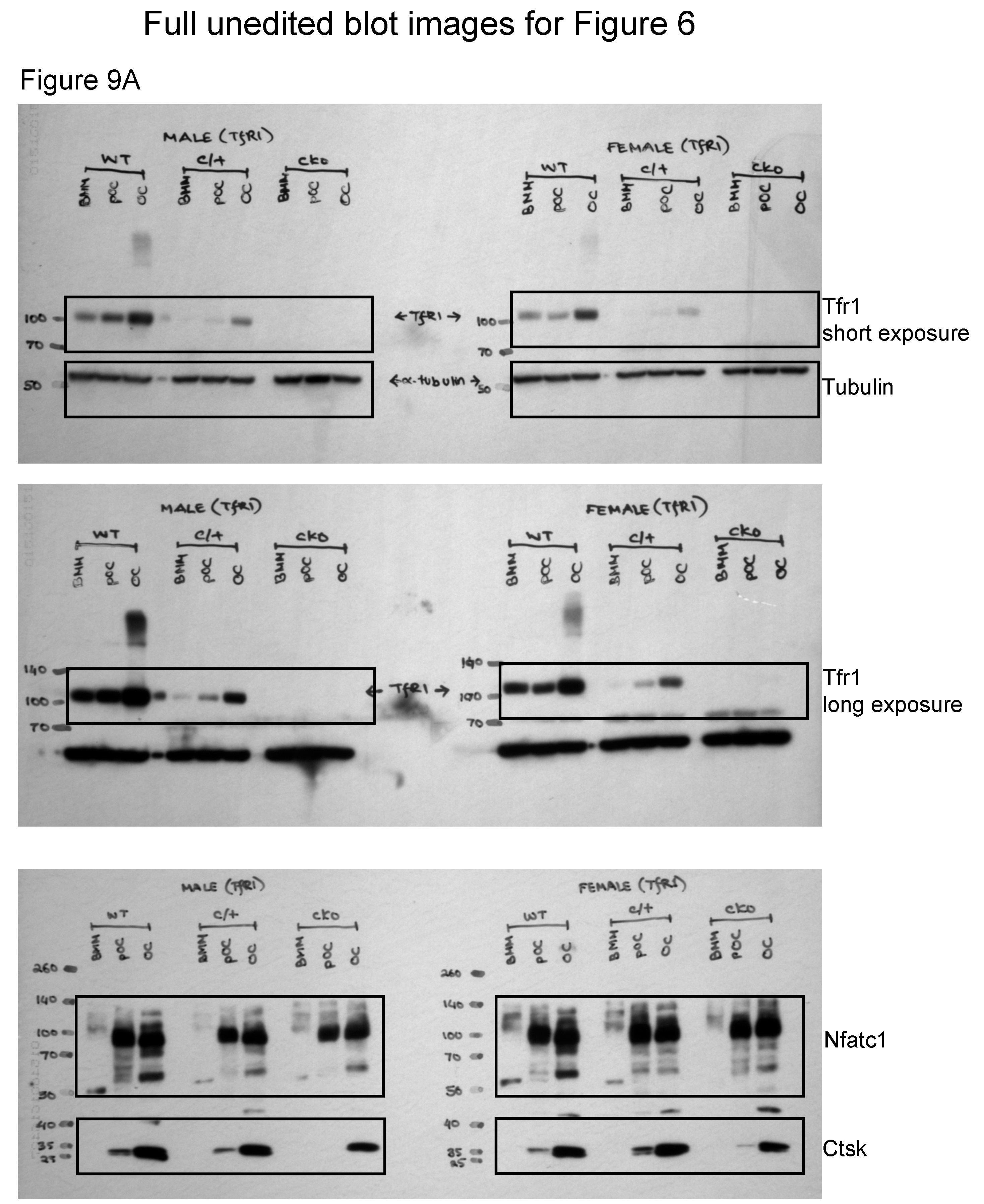

Supplement: Figure 4—source data 2. [file elife-73539-fig4-data2.tif]

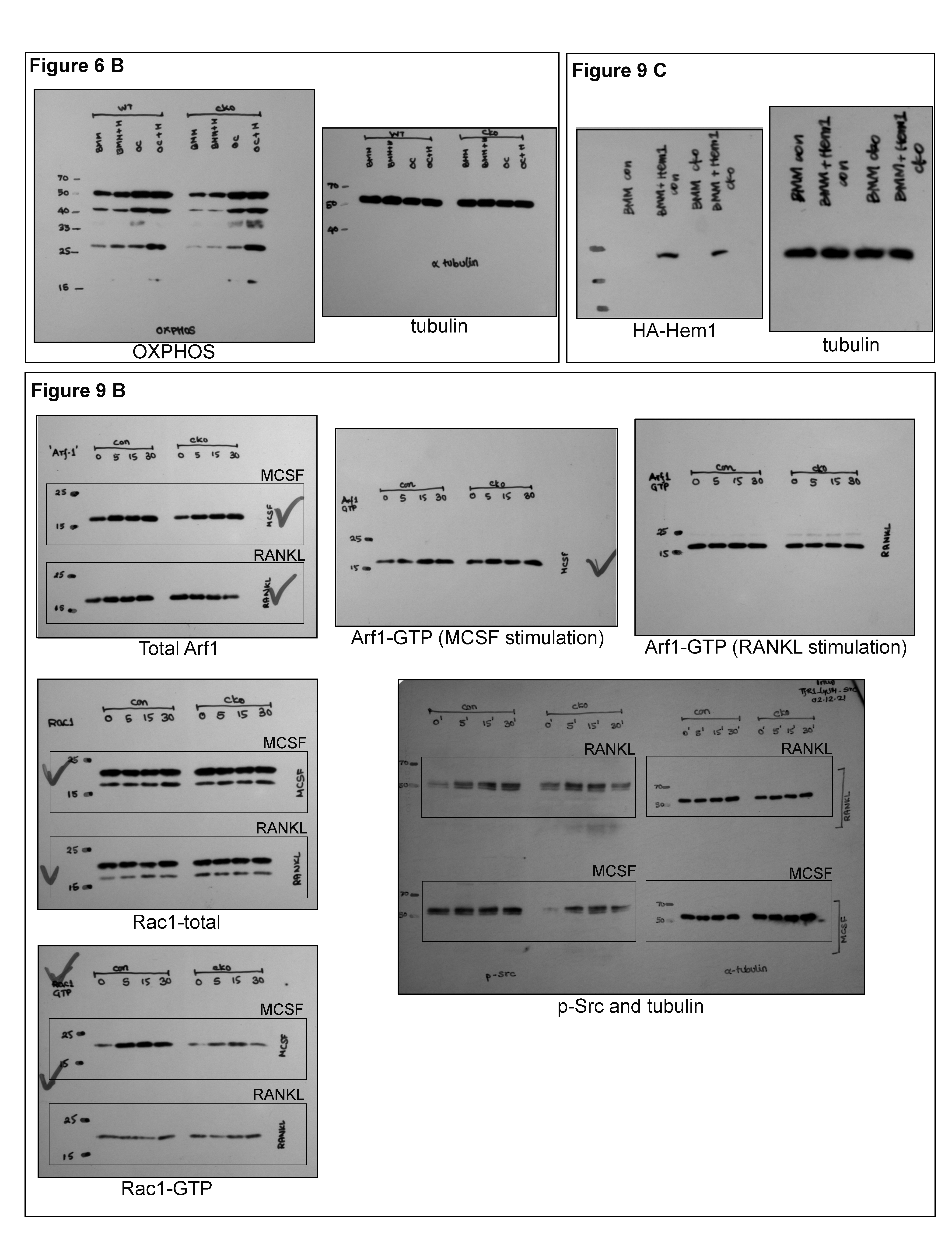

Supplement: Figure 6—source data 1. [file elife-73539-fig6-data1.tif]
