# Supplementary material for: Transferrin receptor 1-mediated iron uptake regulates bone mass in mice via osteoclast mitochondria and cytoskeleton
Source: eLife. 2022 Jun 27;11:e73539. doi: 10.7554/eLife.73539 (PMC9352353; doi:10.7554/eLife.73539)
Supplement: Supplementary file 5. [file elife-73539-supp5.docx]

**Suppl Table 7. Changes of cytoskeletal proteins in TfR1-null osteoclast lineage cells.**

**BMM**

| Gene Symbol | Fold change | p-value |
| --- | --- | --- |
| Cttn | -1.43286 | 0.0005787 |
| Flnb | 1.84547 | 6.49E-05 |

**pOC**

| Gene Symbol | Fold change | p-value |
| --- | --- | --- |
| Mapre1 | -1.18971 | 0.0005099 |
| Rap2b | 1.20116 | 0.0037813 |
| Flnb | 1.37144 | 0.0001004 |

**OC**

**Down No change Up**

| Gene Symbol | Fold change | p-value | Gene Symbol | Fold change | p-value | Gene Symbol | Fold change | p-value |
| --- | --- | --- | --- | --- | --- | --- | --- | --- |
| Myh14 | -2.341 | 0.004486 | Vcl | -1.188 | 0.01273 | Flnb | 1.239 | 0.0008791 |
| Rap1a | -1.841 | 0.006630 | Dock5 | -1.1698 | 0.003040 | Vav3 | 1.267 | 0.002373 |
| Itgb5 | -1.771 | 0.009642 | Rap2c | -1.1687 | 0.02217 | Arfgap1 | 1.270 | 0.002831 |
| Arf6 | -1.666 | 0.004343 | Arpc5 | -1.1515 | 0.02533 | Cfl1 | 1.286 | 0.0001049 |
| Arpc5l | -1.6562 | 0.0005538 | Git1 | -1.1292 | 0.001233 | Rock1 | 1.300 | 0.0002146 |
| HSPC300 | -1.636 | 0.0008513 | Pak1 | -1.1205 | 0.01599 | Arhgef7 | 1.364 | 0.004932 |
| Arf4 | -1.570 | 0.0033280 | Hdac1 | -1.102 | 0.01036 | Abi1 | 1.375 | 0.006532 |
| Cyfip2 | -1.527 | 0.0007909 | Cdc42 | -1.073 | 0.02226 | Mapre1 | 1.397 | 9.19E-05 |
| Cyfip1 | -1.464 | 0.0010528 | Rac1 | 1.067 | 0.3174 | Tln2 | 1.405 | 0.003031 |
| Flna | -1.451 | 0.0004968 | Syk | 1.070 | 0.02148 | Rac2 | 1.581 | 0.0005626 |
| Arfgef2 | -1.445 | 0.0001426 | Arhgef7 | 1.078 | 0.01906 | Wasf2 | 1.620 | 7.17E-05 |
| Rap1b | -1.397 | 0.0054647 | Pxn | 1.110 | 0.02035 | Was | 1.687 | 1.10E-05 |
| Actn1 | -1.393 | 3.74E-05 | Itgb2 | 1.127 | 0.007875 | Arfgap3 | 1.761 | 0.0001522 |
| Arf5 | -1.380 | 0.011386 | Rock2 | 1.131 | 0.004535 | Arfgap2 | 1.877 | 6.03E-06 |
| Actn4 | -1.365 | 0.0005075 | Rapgef6 | 1.181 | 0.01708 | Cttn | 1.921 | 0.0001745 |
| Arpc2 | -1.353 | 0.0002935 |  |  |  | Cfl2 | 2.154 | 0.02121 |
| Hdac2 | -1.341 | 0.0019720 |  |  |  | Hdac6 | 2.350 | 1.31E-05 |
| Arf1 | -1.331 | 0.0023868 |  |  |  |  |  |  |
| Rap2b | -1.309 | 0.001288 |  |  |  |  |  |  |
| Nckap1l | -1.307 | 0.003751 |  |  |  |  |  |  |
| Rapgef1 | -1.296 | 0.01446 |  |  |  |  |  |  |
| Rap1gds1 | -1.234 | 0.006664 |  |  |  |  |  |  |
| Src | -1.229 | 0.004918 |  |  |  |  |  |  |
| Arpc4 | -1.211 | 0.001957 |  |  |  |  |  |  |
| Itgb3 | -1.201 | 0.05545 |  |  |  |  |  |  |
